# Competition between In-Plane vs Above-Plane Configurations of Water with Aromatic Molecules: Non-Covalent Interactions in 1,4-Naphthoquinone-(H_2_O)_1–3_ Complexes

**DOI:** 10.1021/acs.jpclett.2c02618

**Published:** 2022-10-06

**Authors:** Shefali Baweja, Sanjana Panchagnula, M. Eugenia Sanz, Luca Evangelisti, Cristóbal Pérez, Channing West, Brooks H. Pate

**Affiliations:** † Department of Chemistry, King’s College London, 7 Trinity Street, London SE1 1DB, United Kingdom; ‡ Department of Chemistry, University of Virginia, Charlottesville, Virginia 22904-4319, United States

## Abstract

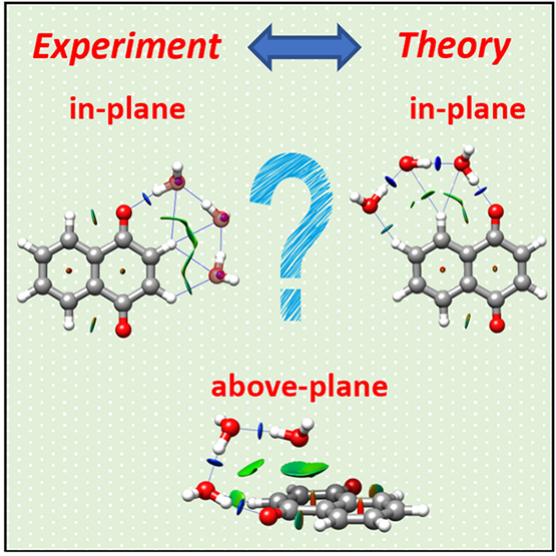

Non-covalent interactions between aromatic molecules and water are fundamental in many chemical and biological processes, and their accurate description is essential to understand molecular relative configurations. Here we present the rotational spectroscopy study of the water complexes of the polycyclic aromatic hydrocarbon 1,4-naphthoquinone (1,4-NQ). In 1,4-NQ-(H_2_O)_1,2_, water molecules bind through O–H···O and C–H···O hydrogen bonds and are located on the plane of 1,4-NQ. For 1,4-NQ-(H_2_O)_3_, in-plane and above-plane water configurations are observed exhibiting O–H···O, C–H···O, and lone pair···π-hole interactions. The observation of different water arrangements for 1,4-NQ-(H_2_O)_3_ allows benchmarking theoretical methods and shows that they have great difficulty in predicting energy orderings due to the strong competition of C–H···O binding with π and π-hole interactions. This study provides important insight into water interactions with aromatic systems and the challenges in their modeling.

An accurate account of non-covalent interactions involving aromatic molecules is paramount to understand aggregation, the behavior of supramolecular materials, and biological outcomes.^1^ The combination of hydrogen bonding, dispersion, dipole–dipole, and repulsive interactions dictate how molecules arrange around one another and drive the emergence of crystal structures, solvation shells, and supramolecular assemblies. Investigating molecular systems with a small number of moieties is fundamental to understand how modest changes in non-covalent interactions influence structural arrangements.^2,3^ Gas-phase data are particularly helpful in this respect, as experimental results can be directly compared with theory since the effect of the environment is removed.

Among small-scale molecular systems, water complexes have been widely studied owing to the relevance of water as universal solvent and its role in biological systems. The configurations and interactions of pure water clusters, from dimer to decamer, and mixtures of water with a variety of partners, have been reported using rotational and infrared spectroscopies.^4−14^ Water is a versatile probe of molecular electron density sites. It can establish hydrogen bonds acting as a hydrogen bond donor or acceptor (through the oxygen lone pairs), and also lone pair-π hole interactions.^15−17^ Therefore, a single water molecule can be involved in several non-covalent interactions, with a solute and with other water molecules, and their combined contributions will determine the overall structure of the complex.

In interactions with aromatic molecules, which usually present more than one binding site, water can adopt different configurations.^18^ For pure, non-substituted aromatic hydrocarbons and for perfluorinated aromatics, water binds to the π electron density and locates itself above the molecular plane of the aromatic.^13,16,17,19−23^ Additional water molecules preferably bind to one another maintaining their above-plane location. If the aromatic molecule contains heteroatoms or functional groups, water binds to them and remains (mostly) on the plane of the aromatic (see examples in refs (13, 22, 24− and 27)). But as the number of water molecules increases, the non-covalent network expands, getting more complex, and competition between in-plane and above-plane configurations of water molecules emerge. The question then arises: how many water molecules are necessary for in-plane and above-plane competition to appear? What are the competing noncovalent interactions involved? Is this competition accurately described by theoretical methods?

We have explored these questions by investigating the stepwise microsolvation of 1,4-naphthoquinone (1,4-NQ), a derivative of naphthalene composed of a benzene fused with a 1,4-benzoquinone ring. 1,4-NQ is an environmental pollutant^28^ and widely used as a scaffold for drug development,^29^ but is of interest here because it presents several regions with different electron densities.^30^ The highest electron density areas in 1,4-NQ are the oxygen lone pairs, while the most electrophilic ones correspond to the hydrogens bound to the double bond of the quinone ring. Perpendicular to the molecular plane, the quinone ring is electrophilic, but the benzene ring maintains its nucleophilic character. These regions can give rise to a variety of interaction networks in complexes of 1,4-NQ with several water molecules.

We studied the complexes of 1,4-NQ with water using broadband rotational spectroscopy,^31^ as this technique is highly sensitive to molecular structure and the analysis of the rotational spectrum provides precise and unambiguous structural determination of molecular systems. Identification of a species is accomplished by observation of many related rotational transitions that follow a unique pattern determined by the species’ mass distribution. The high resolution of rotational spectroscopy allows observation of transitions from different coexisting species without overlap. The use of broadband operation enables recording large sections of the spectrum and hence facilitates identification of spectral patterns.

The rotational spectrum of 1,4-NQ with water was recorded in the 2–8 GHz frequency range using broadband rotational spectrometers at the University of Virginia and King’s College London described previously.^10,32,33^ Experimental details are provided in the Supporting Information (SI). Intense lines belonging to bare 1,4-NQ^30^ and water clusters, including water dimer, hexamers, heptamers, and nonamers,^4,9,10,12^ were observed in the spectrum and removed. Before analyzing the spectrum, we mapped the configurational landscape of complexes of 1,4-NQ with up to four water molecules using Grimme’s XTB program suite, a semiempirical tight binding method.^34,35^ The structures of the predicted isomers were optimized by running calculations at B3LYP^36,37^-D3BJ^38,39^ and MP2^40^ levels of theory with the 6-311++G(d,p)^41,42^ basis set using Gaussian.^43^ Harmonic frequency calculations were performed on the optimized structures to ensure that they were true minima in the potential energy surface. The results (see SI) show a significant rise in the number of possible low-energy isomers as the number of water molecules, and the possible arrangements, increases.

Guided by the spectral patterns predicted from computational calculations, we identified one isomer of 1,4-NQ-H_2_O, two isomers of 1,4-NQ-(H_2_O)_2_, and three isomers of 1,4-NQ-(H_2_O)_3_ in the rotational spectrum, some of them using PGOPHER.^44,45^ The observed species were matched to predicted structures from the close values of experimental and theoretical rotational constants (see Tables 1, 2 and details of the spectral analysis and assignment in the SI). The relative intensities of the *a*-, *b*-, and *c*-type transitions were also consistent with expectations from the theoretical dipole moment components. Further confirmation of our assignment was provided by the observation of the water ^18^O isotopologues of complexes **1w-1**, **2w-1**, **2w-2**, and **3w-1** at the expected frequency shifts, after conducting additional experiments using mixtures of H_2_^16^O and H_2_^18^O. From the rotational constants of the singly substituted ^18^O species, and using Kraitchman’s equations,^46^ we determined the positions of the water oxygens of the complexes (see SI). Their agreement with the theoretical structures is shown in Figure 1. The lower intensity of the spectra of **3w-3** and **3w-4** prevented observation of their ^18^O isotopologues.

**Table 1:** Experimental Spectroscopic Constants for 1,4-NQ-(H_2_O)_1,2_ Complexes

	**1w-1**			**2w-1**			**2w-2**		
**Parameter**	**Experimental**	**MP2**	**B3LYP**	**Experimental**	**MP2**	**B3LYP**	**Experimental**	**MP2**	**B3LYP**
*A*^a^ (MHz)	1210.09271(21)^g^	1203.5	1212.5	1042.60124(45)	1037.1	1046.0	965.35847(34)	966.5	972.3
*B* (MHz)	583.45149(13)	585.6	593.0	391.30769(16)	391.5	398.6	448.52008(11)	450.1	455.3
*C* (MHz)	393.98342(10)	394.4	398.3	284.87127(16)	284.6	288.9	306.77777(12)	308.0	310.8
*Δ*_*J*_ (kHz)	0.02537(95)	0.023	0.0187	0.0059(12)	0.009	0.008	0.00916(57)	0.017	0.016
*Δ*_*JK*_ (kHz)	0.07456(29)	0.055	0.0693	0.2359(65)	0.165	0.122	0.1890(56)	0.134	0.184
*Δ*_*K*_ (kHz)	–0.0382(55)	–0.009	–0.029	–0.145(16)	–0.110	–0.075	–0.128(12)	–0.111	–0.158
*δ*_*J*_ (kHz)	0.00669(45)	0.007	0.0067	–	0.002	0.002	–	0.005	0.005
*δ*_*K*_ (kHz)	0.0890(40)	0.064	0.0611	0.084(18)	0.098	0.075	0.0553(87)	0.093	0.116
*P*_c_^b^ (uÅ^2^)	0.54167(19)	0.8	0.1	1.09053(57)	1.2	0.9	1.45312(36)	2.4	1.8
|*μ*_*a*_|^c^ (D)	y	1.5	1.8	y	1.4	1.7	y	0.4	0.2
|*μ*_*b*_| (D)	y; *μ*_*b*_*> μ*_*a*_	2.4	2.6	y; *μ*_*b*_*> μ*_*a*_	2.9	3.0	y; *μ*_*b*_*> μ*_*a*_	1.3	1.1
|*μ*_*c*_| (D)	n	0.8	0.5	n	0.1	0.1	n	0.2	0.1
σ^d^ (kHz)	3.6	–	–	7.2	–	–	4.7	–	–
N^e^	130	–	–	97	–	–	106	–	–
Δ*E*_0_^f^ (kJ mol^–1^)	–	0.0	0.0	–	0.0	0.0	–	1.5	2.5

a *A*, *B* and *C* are the rotational constants; Δ_*J*,_ Δ_*JK*,_ Δ_*K*,_*δ*_*J*_, and *δ*_*K*_ are the quartic centrifugal distortion constants.

b Planar moment of inertia *P*_*c*_ = ∑_i_*m*_i_*c*_i_^2^.

c Yes (y) or no (n) observation of *a*-, *b*-, and *c*-type transitions, and absolute theoretical values of the dipole moment components along the principal inertial axis system.

d rms deviation of the fit.

e Number of fitted transitions.

f Zero-point corrected energies.

g Standard error in parentheses in units of the last digit.

**Table 2:** Experimental Spectroscopic Constants for 1,4-NQ-(H_2_O)_3_ Complexes

	**3w-1**			**3w-3**			**3w-4**		
**Parameter**	**Experimental**	**MP2**	**B3LYP**	**Experimental**	**MP2**	**B3LYP**	**Experimental**	**MP2**	**B3LYP**
*A*^a^ (MHz)	827.73556(37)^g^	825.7	836.7	807.78287(55)	804.2	816.1	763.99060(67)	764.8	778.3
*B* (MHz)	314.314215(74)	315.9	318.5	381.67975(29)	400.8	389.0	328.89636(26)	325.9	330.5
*C* (MHz)	231.011719(60)	230.5	233.0	320.72522(15)	331.4	326.6	234.68896(20)	231.9	234.9
*Δ*_*J*_ (kHz)	0.02746(29)	0.018	0.022	0.0691(14)	0.033	0.050	0.02204(84)	0.021	0.018
*Δ*_*JK*_ (kHz)	–0.0616(29)	0.023	0.046	0.093(13)	0.115	0.163	0.240(12)	0.082	0.081
*Δ*_*K*_ (kHz)	0.299(19)	0.174	0.208	–	0.006	0.095	–	0.014	0.017
*δ*_*J*_ (kHz)	0.00586(16)	0.005	0.006	0.01534(73)	0.006	0.010	–	0.005	0.004
*δ*_*K*_ (kHz)	–	0.050	0.053	–	0.019	0.159	0.093(16)	0.090	0.082
*P*_c_^b^ (uÅ^2^)	15.3860(10)	9.7	10.9	186.9954(7)	182.2	185.5	22.3450(10)	16.1	13.5
|*μ*_*a*_|^c^ (D)	y	1.2	1.6	n	1.0	0.8	n	0.1	0.1
|*μ*_*b*_| (D)	y; *μ*_*b*_*> μ*_*a*_	3.4	3.3	y	2.2	2.5	y	2.5	2.2
|*μ*_*c*_| (D)	n	0.6	0.5	n	0.1	0.4	n	0.7	0.6
σ^d^ (kHz)	4.1	–	–	4.3	–	–	6.2	–	–
N^e^	159	–	–	39	–	–	49	–	–
Δ*E*_0_^f^ (kJ mol^–1^)	–	9.9	0.0	–	4.9	1.4	–	12.5	2.1

a *A*, *B*, and *C* are the rotational constants; Δ_*J*,_ Δ_*JK*,_ Δ_*K*,_*δ*_*J*_, and *δ*_*K*_ are the quartic centrifugal distortion constants.

b Planar moment of inertia *P*_*c*_ = ∑_i_*m*_i_*c*_i_^2^.

c Yes (y) or no (n) observation of *a*-, *b*-, and *c*-type transitions, and absolute theoretical values of the dipole moment components along the principal inertial axis system.

d rms deviation of the fit.

e Number of fitted transitions.

f Zero-point corrected energies.

g Standard error in parentheses in units of the last digit.

**Figure 1:**
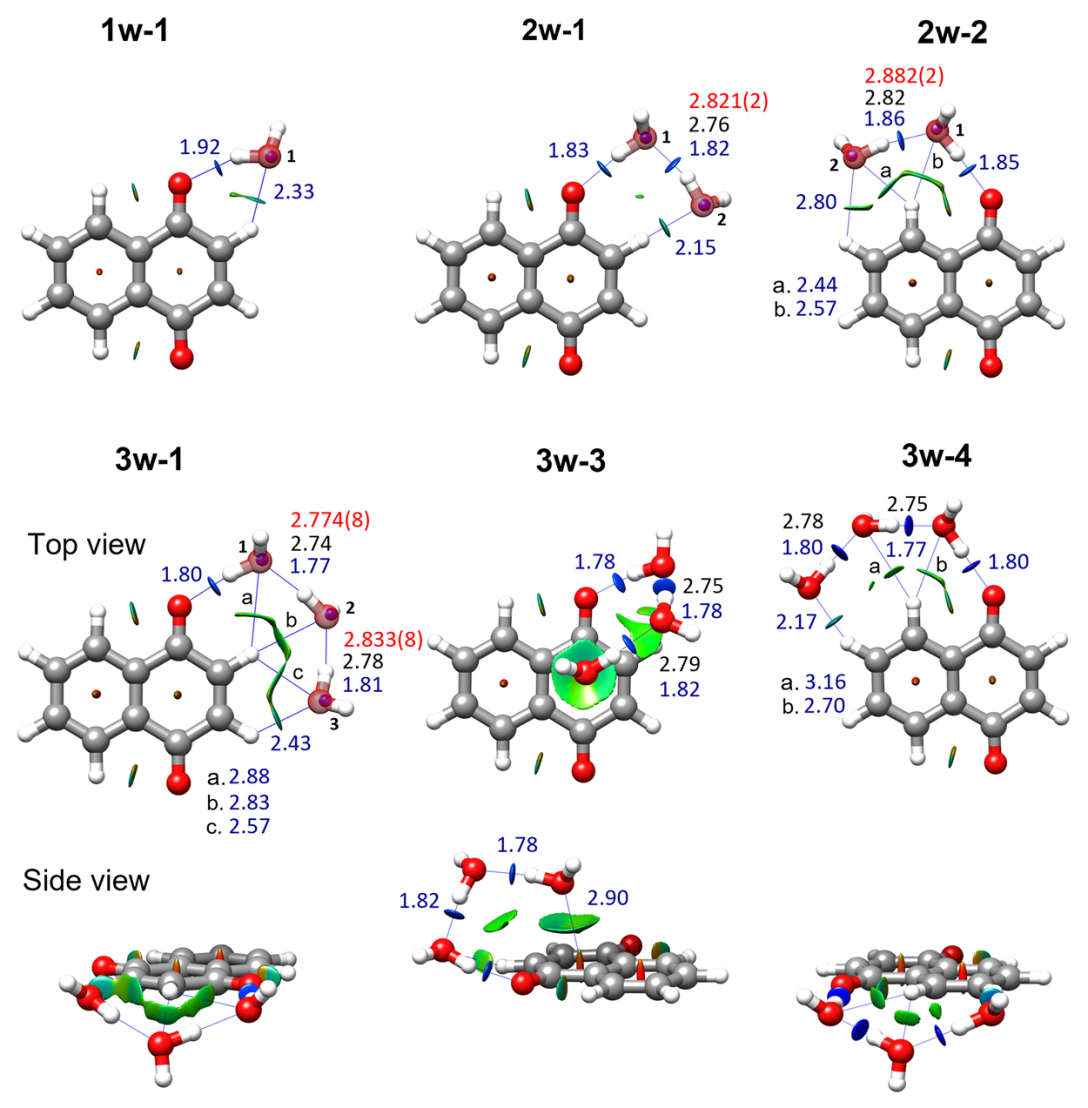
B3LYP-D3BJ structures of the observed 1,4-NQ-(H_2_O)_1–3_ complexes showing their NCI isosurfaces (*s* = 0.5) for values of sign(λ_2_)ρ from −0.025 to +0.025 au. Blue indicates strong attractive interaction; green indicates weak attractive interaction; and red indicates strong repulsive interaction. The experimental position of the oxygen atoms is represented by blue balls. Hydrogen bonds are indicated in blue, and O–O distances are in black (theoretical) and red (experimental r_s_).

We have estimated the relative abundances of the complexes of 1,4-NQ with two and three water molecules from careful measurements of the relative intensities of common *b*-type transitions, and using the MP2 dipole moment predictions for NQ-(H_2_O)_2_ and the B3LYP-D3BJ for NQ-(H_2_O)_3_, as on average those were the calculations yielding rotational constants closer to experimental ones. We found that the relative abundances are **2w-1:2w-2** = 1.4:1 and **3w-1:3w-3:3w-4** = 12:1.5:1.

Initial data on the arrangement of the water molecules can be obtained from the experimental values of the planar moment of inertia *P*_*c*_ = ∑_i_*m*_i_*c*_i_^2^, which inform on the mass distribution out of the *ab* inertial plane of each complex. For complexes **1w-1**, **2w-1**, and **2w-2**, the *P*_*c*_ values of the parent and ^18^O species are nearly invariant (Tables 1, S3, S6). This indicates that the substituted oxygen atoms are on the *ab* plane, which coincides with the plane of 1,4-NQ. The *P*_*c*_ values of these complexes are slightly larger than the *P*_*c*_ value of 1,4-NQ, 0.21342(10) uÅ^2^,^30^ owing to the out-of-plane arrangement of one and two hydrogen atoms in the 1,4-NQ-H_2_O and 1,4-NQ-(H_2_O)_2_ complexes, respectively. For **3w-1**, **3w-3**, and **3w-4**, the values of *P*_*c*_ increase substantially, indicating that not all the water molecules are on the 1,4-NQ plane (Table 2). For **3w-1**, the changes in *P*_*c*_ for the ^18^O isotopologues (Table S12) show that the off-plane contribution arises from the water in the middle of the chain. A similar arrangement can be expected for **3w-4**, with a slightly larger value of *P*_*c*_ that can be attributed to a more pronounced out-of-plane configuration of its water molecules. For **3w-3**, the *P*_*c*_ is about 10 times higher than those for **3w-1** and **3w-4**, indicative of even larger off-plane water contributions.

The configurations adopted by the complexes are determined by a network of intermolecular interactions involving water and two or more electron density regions of 1,4-NQ. Interactions are visualized in Figure 1 using the NCI method,^47^ which considers electron density and its derivatives to classify non-covalent interactions. In all complexes, one water molecule binds to one of the oxygen lone pairs of 1,4-NQ through an O–H···O hydrogen bond. Additional water molecules bind to one another through further O–H···O bonds and establish C–H···O hydrogen bonds with 1,4-NQ. The pairs **2w-1**/**2w-2** and **3w-1**/**3w-4** have comparable topologies, with water molecules arranged as open chains on either side of 1,4-NQ, mostly in-plane as discussed above. Topology and interactions are remarkably different for **3w-3**, where all water molecules are above 1,4-NQ’s plane and one of them is located directly above the quinone ring. The quinone ring is a π-hole, a region of lower electron density perpendicular to the molecular frame,^48^ and thus water binds to it through the oxygen lone pair establishing a lone pair-π hole (lp-π) interaction. This is depicted as a large greenish isosurface, very different from the blue pill-like appearance of the O–H···O bonds, indicating its weaker and less directional character.

From the isotopologue data we could determine the *r*_s_ O–O distances (Figure 1 and SI). Their values are very similar to those determined for other water complexes involving a ketone and open chains of up to three water molecules.^49−53^ As the number of water molecules increase, hydrogen bond lengths and O–O distances decrease due to cooperativity effects.^54^

However, cooperativity is reduced in the case of **3w-1** and **3w-4**, which have τ(OOOO) dihedral angles of −31.6° and 41.7°, respectively. To our knowledge, this large departure from coplanarity had only been observed before in the complexes of formamide and ethyl carbamate with three molecules of water^55,56^ (where the third water molecule binds to a N atom through a N–H···O bond), and shows the adaptability of water molecules to bind to substrates. This is also reflected in the variable values of the angle ∠C_NQ_O_NQ_H_w_ (Figure 1), moving from 116.9° to 131.8° to 123.8° in going from **1w-1** to **2w-1** to **3w-1**, while being 140.3° and 142.2° in **2w-2** and **3w-3**, respectively.

The fine balance between the various intermolecular hydrogen bonding, and in-plane vs above-plane configurations, is exposed in the 1,4-NQ-(H_2_O)_3_ complexes. In 1,4-NQ-(H_2_O)_1,2_, O–H···O hydrogen bonds reinforced by C–H···O bonds dominate, and in-plane configurations are clearly preferred (Figures S2, S3). In 1,4-NQ-(H_2_O)_3_, C–H···O binding competes with π and π-hole interactions involving the benzene and quinone rings, respectively, and configurations where water molecules are located above 1,4-NQ’s plane become relevant (Figure S4). In 1,4-NQ-(H_2_O)_4_, the shift in preferences is complete and above-plane configurations dominate (Figure S5).

Modeling the array of competing interactions is challenging for theoretical methods. All observed complexes were minima predicted by XTB, but as expected, the energy ordering differed substantially from that given by quantum chemical calculations. B3LYP-D3BJ and MP2 calculations predict the same energy ordering for the lower-energy isomers of 1,4-NQ-(H_2_O)_1,2,4_. However, large differences arise for 1,4-NQ-(H_2_O)_3_ (Tables 2, S7, S8). Isomer **3w-1**, the global minimum by B3LYP-D3BJ calculations, is predicted to lie at an astonishing 9.9 kJ mol^–1^ by MP2. Similarly, **3w-4**, the other isomer with in-plane interactions, goes from being predicted 2.1 kJ mol^–1^ above the global minimum by B3LYP to 12.5 kJ mol^–1^ by MP2. All MP2 low-energy isomers show water molecules in above-plane configurations. It is known that MP2 has problems in describing interaction energies due to a poor description of dispersion.^57^ This can translate in large deviations of MP2 structures from experimental ones,^2,58,59^ but it is not the case for 1,4-NQ-(H_2_O)_3_ where MP2 and experimental rotational constants are close, and show average differences of 0.3%, 0.7%, and 2.9% for **3w-1**, **3w-4**, and **3w-3**, respectively. The main issue with MP2 calculations lies on the predicted energy ordering, which cannot be reconciled with our relative abundance observations of **3w-1:3w-3:3w-4** = 12:1.5:1.

The above discrepancies prompted us to carry out additional geometry optimizations of the lower-energy isomers of 1,4-NQ-(H_2_O)_3_ using B3LYP-D4, wB97X-D3, and B2PLYP methods with the def2-TZVP basis set, and the RI-MP2/aug-cc-pVTZ level of theory using ORCA.^60,61^ We also performed single-point energy calculations using the explicitly correlated MP2-F12 theory^62^ on the MP2/6-311++G(d,p) and RI-MP2/aug-cc-pVTZ structures. All theory methods perform similarly in predicting the isomers’ structures, except wB97X-D3, which shows large deviations between experimental and theoretical rotational constants (up to 6.2%, Table S13). MP2 methods show larger deviations for **3w-3**. However, energy orderings change a lot among different methods (Tables S7–S11). Using the aug-cc-pVTZ basis set reduces the difference between MP2 relative energies and those from other methods. Including the F12 correlation reduces the difference further for both basis sets. Both RI-MP2-F12 calculations predict almost the same energy ordering of the isomers, so we will just refer to RI-MP2-F12 in the discussion below.

The predicted global minimum varies among methods, but once F12 corrections are included, all methods predict **3w-1**, **3w-3**, and **3w-4** to be among the four lowest-energy isomers. Open chain **3w-1**, the most abundant isomer observed, is only predicted as a global minimum by B3LYP-D3BJ; the other methods, except MP2, predict it as the third or fourth in the energy ordering. Open loop **3w-2** is predicted as the global minimum by MP2, RI-MP2-F12, B3LYP-D4, and B2PLYP. This isomer was repeatedly searched for in our spectrum but without success. **3w-2** is predicted to be lower in energy than **3w-3**, but its lower dipole moment components decrease its spectral intensity, likely preventing its observation.

The larger discrepancies among lower-energy predicted complexes involve **3w-4** and **3w-8**. **3w-8**, with the water trimer located above 1,4-NQ, is predicted as the global minimum by wB97X-D3 and B2PLYP, second in energy by RI-MP2-F12 calculations, and fifth and eighth by B3LYP-D4 and B3LYP-D3BJ, respectively. This isomer has also been repeatedly searched for in our spectrum, but it has not been observed. Open chain **3w-4**, which we have observed, is predicted to be fourth and fifth in energy by B3LYP-D3BJ and B2PLYP, respectively. Other methods predicted it to be even higher in the energy ordering.

In conclusion, theoretical methods have great difficulty in predicting energy ordering where there is strong competition among non-covalent interactions leading to different configurations of water molecules around an aromatic substrate. For 1,4-NQ-(H_2_O)_3_, this translates into wildly different relative energy predictions for open chain, open loop, and water trimer arrangements in in-plane vs above-plane configurations, involving competition between C–H···O binding with π and π-hole interactions.

The several binding sites in 1,4-NQ make it an ideal substrate for scaffolding applications and explain the widespread use of substituents to tune its different electron density areas. In this context, π and lp-π interactions are likely to play important roles. The lp-π interaction had been only reported in a handful of complexes in the gas phase, all involving fully fluorinated alkenes or aromatics,^15−17,63^ but its observation in 1,4-NQ indicates that it may be more common. This interaction is known to take place in biological structures such as Z-DNA, and in proteins involving the water oxygen and aromatic residues.^64^

The detection of several complexes of 1,4-NQ-(H_2_O)_3_ allowed benchmarking of computational methods and highlighted the challenges in describing the interactions of aromatic molecules with water. Similar issues in modeling competing non-covalent interactions can be expected to arise for complexes of other aromatic molecules with water. A combined experimental and theoretical approach is necessary to advance our understanding and develop improved models to characterize these systems.

## Supporting Information Available

Computational methods, experimental methods, details of spectroscopic analyses, lists of rotational transitions and Cartesian coordinates of observed complexes (PDF)
