# Supplementary material for: Competition between In-Plane vs Above-Plane Configurations of Water with Aromatic Molecules: Non-Covalent Interactions in 1,4-Naphthoquinone-(H2O)1–3 Complexes
Source: J Phys Chem Lett. 2022 Oct 6;13(40):9510–6. doi: 10.1021/acs.jpclett.2c02618 (PMC9575146; doi:10.1021/acs.jpclett.2c02618)
Supplement: Supplementary file 1 — jz2c02618_si_001.pdf [file jz2c02618_si_001.pdf]

Competition between In-plane vs. Above-Plane Configurations of Water with Aromatic Molecules: Non-covalent Interactions in 1,4-Naphthoquinone-$(H_2O)_{1-3}$ Complexes

Shefali Baweja,[a] Sanjana Panchagnula,[a] M. Eugenia Sanz,*[a] Luca Evangelisti,[b],[c] Cristóbal Pérez,[b,d] Channing West, [b] and Brooks H. Pate[b]

[a] Department of Chemistry, King's College London, London, SE1 1DB (United Kingdom)
E-mail: maria.sanz@kcl.ac.uk
[b] Department of Chemistry, University of Virginia, Charlottesville, VA 22904-4319, USA
[c] Present address: Department of Chemistry "G. Ciamician", University of Bologna, Via S. Alberto 163, Ravenna, 48100, Italy.
[d] Present address: Departmento de Química Física e Inorgánica, Facultad de Ciencias-I.U. CINQUIMA, Universidad de Valladolid, E-47011 Valladolid, Spain.

Supplementary Information

Table of Contents

1. **Computational methods**
2. **Experimental methods**
3. **Spectral analysis and assignment**
4. **Broadband rotational spectrum**

   Figure S1. Left: section of the broadband rotational spectra of 1,4-NQ-water showing transitions from the observed isomers. Right: $6_{1,6} \leftarrow 5_{0,5}$ transition of the three observed isomers of 1,4-NQ-(H_2O)$_3$.

5. **1,4-Naphthoquinone-H_2O**

   Figure S2. Optimised structures of 1,4-NQ-H_2O isomers within 1000 cm^{-1}.
   Table S1. MP2/6-311++G(d,p) spectroscopic parameters for 1,4-NQ-H_2O complexes.
   Table S2. B3LYP-D3BJ/6-311++G(d,p) spectroscopic parameters for 1,4-NQ-H_2O complexes.
   Table S3. Experimental spectroscopic parameters of the $H_2{}^{18}O$ isotopologue of isomer **1w-1** of 1,4-NQ-H_2O.

6. **1,4-Naphthoquinone-(H_2O)$_2$**

   Figure S3. Optimised structures of 1,4-NQ-(H_2O)$_2$ isomers within 1000 cm^{-1}.
   Table S4. MP2/6-311++G(d,p) spectroscopic parameters for 1,4-NQ-(H_2O)$_2$ complexes.
   Table S5. B3LYP-D3BJ/6-311++G(d,p) spectroscopic parameters for 1,4-NQ-(H_2O)$_2$ complexes.
   Table S6. Experimental spectroscopic parameters for the $H_2{}^{18}O$ isotopologues of 1,4-NQ-(H_2O)$_2$ complexes.

7. **1,4-Naphthoquinone-(H_2O)$_3$**

   Figure S4. Optimised structures of 1,4-NQ-(H_2O)$_3$ isomers within 1000 cm^{-1}.
   Table S7. MP2/6-311++G(d,p) spectroscopic parameters for 1,4-NQ-(H_2O)$_3$ complexes.
   Table S8. B3LYP-D3BJ/6-311++G(d,p) spectroscopic parameters for 1,4-NQ-(H_2O)$_3$ complexes.
   Table S9. B3LYP-D4/def2-TZVP spectroscopic parameters for 1,4-NQ-(H_2O)$_3$ complexes.
   Table S10. WB97X-D3/def2-TZVP spectroscopic parameters for 1,4-NQ-(H_2O)$_3$ complexes.
   Table S11. B2PLYP-D3BJ/def2-TZVP spectroscopic parameters for 1,4-NQ-(H_2O)$_3$ complexes.
   Table S12. Experimental spectroscopic parameters for the $H_2{}^{18}O$ isotopologues of the isomer **3w-1** of 1,4-NQ-(H_2O)$_3$.

8. **1,4-Naphthoquinone-(H_2O)$_4$**

   Figure S5. Optimised structures of 1,4-NQ-(H_2O)$_4$ isomers within 600 cm^{-1}.
   Table S13. Comparison of experimental and theoretical rotational constants for the observed 1,4-NQ-(H_2O)$_3$ complexes ($A_{calc} - A_{exp}$)/A_{exp} × 100%.
   Table S14. MP2/6-311++G(d,p) spectroscopic parameters for the 1,4-NQ-(H_2O)$_4$ complexes.
   Table S15. B3LYP-D3BJ/6-311++G(d,p) spectroscopic parameters for the 1,4-NQ-(H_2O)$_4$ complexes.

9. **Structural analysis**

   Table S16. Substitution (r_s) and B3LYP-D3BJ/6-311++G(d,p) coordinates in Å of the oxygen atoms of the water molecules in 1,4-NQ-(H_2O)$_{1-3}$ complexes.
   Figure S7. Plots of the reduced density gradient (RDG) versus sign(λ_2)ρ for the observed isomers of 1,4-NQ-H_2O, 1,4-NQ-(H_2O)$_2$ and 1,4-NQ-(H_2O)$_3$.

10. **Tables of frequencies**

    Tables S17-S34. Measured frequencies and residuals (in MHz) for the rotational transitions of all observed isomers of 1,4-NQ-(H_2O)$_{1-3}$ and their isotopologues.

11. **Theoretical cartesian coordinates of observed complexes**

    Tables S35-S40. Cartesian coordinates of the observed isomers of 1,4-NQ-(H_2O)$_{1-3}$ from B3LYP-D3BJ/3-611++G(d,p).

1. Computational Methods

The configurational space of the complexes of 1,4-naphthoquinone (1,4-NQ) with one, two, three and four water complexes was explored using Grimme's XTB program suite[1,2]. The structures of the predicted configurations were optimised by running calculations at B3LYP-D3BJ and MP2 levels of theory with the 6-311++G(d,p) basis set using Gaussian[3]. Harmonic frequency calculations were performed on the optimised structures to ensure that they were true minima in the potential energy surface. The rotational constants, dipole moment components and relative energies for the lower-energy complexes are collected in Tables S1-S2 (1,4-NQ-H_2O), S4-S5 (1,4-NQ-$(H_2O)_2$), S7-S11 (1,4-NQ-$(H_2O)_3$) and S13-S14 (1,4-NQ-$(H_2O)_4$). The counterpoise method[4], including fragment relaxation[5], was applied to account for basis set superposition errors (BSSE) in the interaction energies of the complexes.

Due to large discrepancies between the relative energies obtained for the 1,4-NQ-$(H_2O)_3$ complexes from B3LYP-D3BJ and MP2 theoretical methods, further calculations at B3LYP-D4, WB97X-D3 and B2PLYP-D3BJ with the def2-TZVP basis set, and the RI-MP2/aug-cc-pVTZ level of theory[6,7] were performed using ORCA[6,7]. Single-point energy calculations using the explicitly correlated MP2-F12 theory[8] on the MP2/6-311++G(d,p) and RI-MP2/aug-cc-pVTZ structures were also carried out. The spectroscopic parameters obtained with all these methods are listed in Tables S8-S11.

The non-covalent interactions (NCI)[9] in the complexes were visualised using Multiwfn[10], a multifunctional wavefunction analyser used to achieve an interactive visualization based on the topology of the electronic densities in the complexes.

2. Experimental Methods

1,4-NQ (97%) was purchased from Sigma-Aldrich and used without further purification. Chirped-pulse Fourier transform microwave spectrometers at King's College London (KCL)[11,12] and University of Virginia (UVA)[13] were used to analyse the spectrum of 1,4-NQ with water. The sample of 1,4-NQ was seeded in neon as a carrier gas (at a backing pressure of 3 bar at UVA, 5 bar at King's) and mixed with water. 1,4-NQ was heated in custom-made heating receptacles attached to the nozzle to 403.15 K to increase its concentration in the gas phase. Water was placed in an external reservoir connected to the injection system. The optimal vaporisation temperature was found by monitoring the intensity of spectra at different temperatures.

The mixture of 1,4-NQ and water was introduced into the vacuum chamber, where it formed a supersonic expansion. In the instrument at KCL, molecular pulses of 1000 µs length were used, as well as 4 µs chirped microwave pulses, which were applied with a delay of 100 µs with respect to the end of the molecular pulse. After the microwave radiation excitation stopped, the molecules relax back to their initial rotational states, emitting free induction decay (FID) relaxation signals that were collected for 20 µs in the time domain and then were converted to the frequency domain via a fast Fourier-transform algorithm. The final spectrum had 3768k FIDs.

The spectrometer at UVA used 4 pulsed nozzles[13] between the two microwave horns. The sample was polarized by eight chirped microwave pulses (4 µs each) back-to-back per gas injection using the fastframe option of the oscilloscope. After each excitation pulse, the molecular emission was collected in the time domain for 40 µs, averaged and subsequently Fourier transformed to yield the frequency domain spectrum. The final spectrum had 760k FIDs. Experiments using pure $H_2{}^{16}O$ and enriched ^{18}O water in proportions of 20:80 $H_2{}^{18}O$:$H_2{}^{16}O$ and 50:50 $H_2{}^{18}O$:$H_2{}^{16}O$ were performed in both spectrometers to observe the ^{18}O isotopologues of the water molecules.

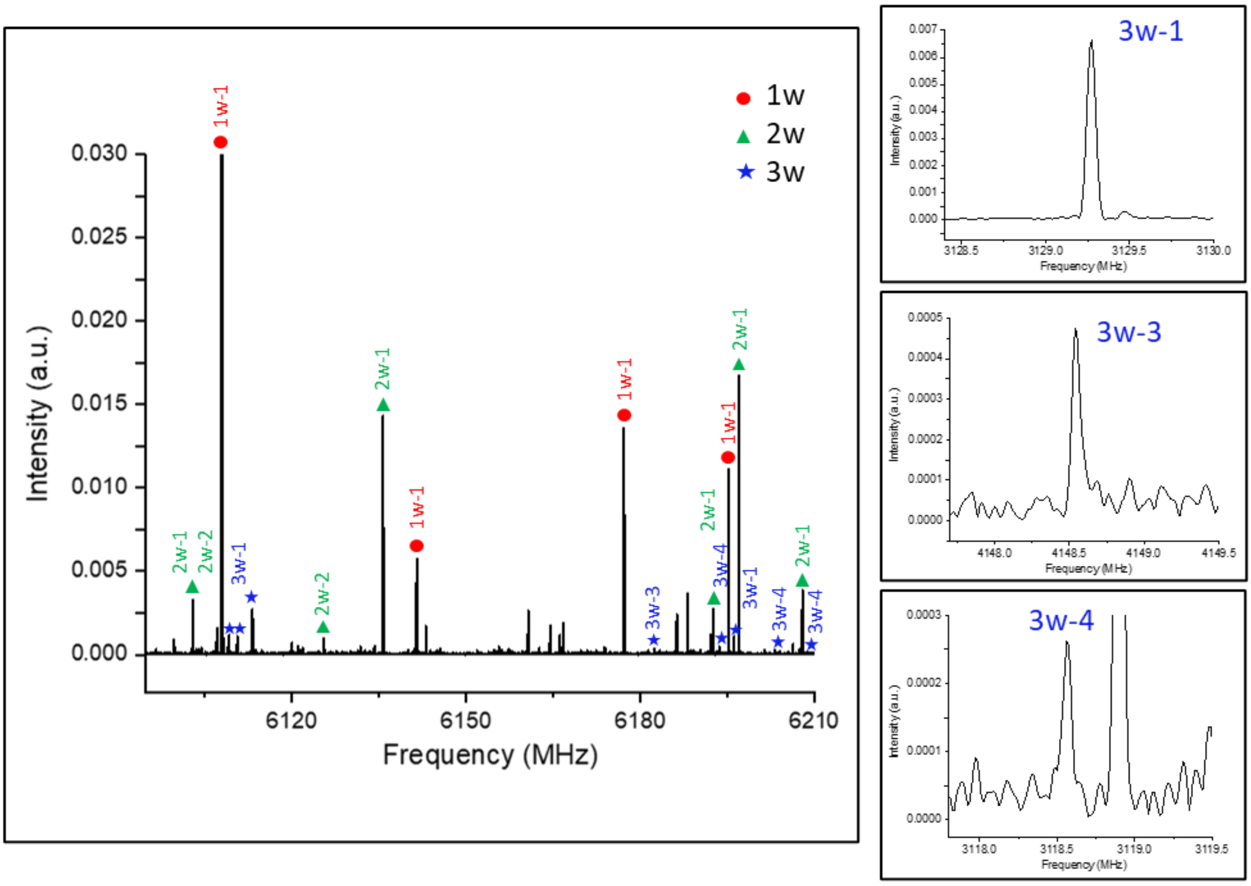

Figure S1. Left: section of the broadband rotational spectra of 1,4-NQ-water showing transitions from the observed isomers. Right: $6_{1,6} \leftarrow 5_{0,5}$ transition of the three observed isomers of 1,4-NQ-$(H_2O)_3$.

3. Spectral Analysis and Assignment

3.1. 1,4-NQ-H_2O

The lower-energy complexes predicted theoretically for 1,4-NQ-H_2O involve the interaction of the water molecule with one of the carbonyl oxygen lone pairs. A configuration where water is above 1,4-NQ is predicted to be much higher in energy.

We started searching for *b*-type transitions for the lowest-energy isomer since μ_b is predicted as its major dipole moment (see Tables 1, S1, S2). A pattern corresponding to R-branch *b*-type transitions of the series $J'_{1,J'} \leftarrow J''_{0,J''}$ and $J'_{0,J'} \leftarrow J''_{1,J''}$ was observed and identified as arising from the lowest-energy isomer **1w-1**. Eventually, *a*- and *b*-type transitions were measured and fitted using Pickett's SPFIT[14]. No *c*-type transitions were observed. This could be due to μ_c being effectively zero due to a large amplitude wagging motion of the water molecule, where the unbound H atom moves above and below the plane of 1,4-NQ. The final experimental rotational and quartic centrifugal distortion constants were determined using Watson's A-reduced Hamiltonian in the I^r representation[15] and are shown in Table 1 along with the theoretical ones. Further confirmation of the observation of **1w-1** was obtained through the assignment of $H_2{}^{18}O$ isotopologue, found at the predicted frequency shifts (see Table S3).

The next isomer in energy, **1w-2**, was then searched for in the spectrum, but it could not be found. The largest dipole moment component for **1w-2** is μ_c, with a value similar to that predicted for **1w-1.** No *c*-type transitions were observed for **1w-1**. If the motion postulated above for **1w-1** also occurs in **1w-2**, it will explain the non-observation of *c*-type transitions. *a*- and *b*-type transitions of **1w-2** were also searched for, but they could not be observed, which may be due to the low values of the μ_a and μ_b components of the dipole moment (Tables S1, S2).

For the next isomer **1w-3**, predictions of its relative energy are surprisingly different, with MP2 predicting it to lie at *ca*. 230 cm^{-1} and B3LYP-D3BJ at *ca*. 1030 cm^{-1}. Both methods predict relatively large μ_a and μ_c dipole moment components. Repeated searches for **1w-3** in our spectrum did not return a positive identification. Therefore we concluded that it is not sufficiently populated.

3.2. 1,4-NQ-$(H_2O)_2$

The lower-energy isomers of 1,4-NQ-$(H_2O)_2$ complexes are predicted to be prolate asymmetric tops and have μ_b as the largest dipole moment component (Tables S4, S5). Therefore, we searched for R-branch *b*-type transitions of the type $J'_{1,J'} \leftarrow J''_{0,J''}$ and $J'_{0,J'} \leftarrow J''_{1,J''}$. Two series of *b*-type transitions corresponding to two different species were found, the second one using the program PGOPHER[16]. Final fits including both *a*- and *b*-type transitions yielded the rotational and centrifugal distortion constants of Table 2. The fits were completed through SPFIT[14] using Watson's A reduced Hamiltonian in the I^r representation[15]. The observed species were unambiguously assigned to isomers **2w-1** and **2w-2** from the comparison between theoretical and experimental rotational constants. The observation of all singly substituted ^{18}O species arising from the water molecules at the predicted frequency shifts (Table S6) confirmed the assignments.

The next isomer in energy, **2w-3**, was searched for but it was not observed, probably because of its low population in our supersonic jet, as it is predicted to lie higher in energy.

The **2w-2** complex was found to be about 1.4 times as intense as **2w-1** from the estimation of relative abundances by careful measurements of the relative intensities of common *b*-type transitions, and using the MP2 dipole moment predictions.

3.3. 1,4-NQ-$(H_2O)_3$

After the removal of lines from 1,4-NQ-H_2O and 1,4-NQ-$(H_2O)_2$ complexes, there were still intense lines in the spectrum. Hence, we searched for the complexes of 1,4-NQ with three water molecules. Looking at the MP2 and B3LYP-D3BJ predictions (Tables S7, S8), there are significant discrepancies in the relative energy ordering of the isomers, and no agreement on the global minimum. For example, the lowest energy complex at B3LYP-D3BJ level, **3w-1**, is predicted to lie at approximately 830 cm^{-1} above the global minimum by MP2.

The major dipole moment component for the lower-energy isomers of 1,4-NQ-$(H_2O)_3$ is *b*-type, and therefore we followed the same strategy of looking for R-branch *b*-type transitions as we did for 1,4-NQ-H_2O and 1,4-NQ-$(H_2O)_2$. We observed three different 1,4-NQ-$(H_2O)_3$ complexes in the spectra, two of them with the aid of PGOPHER[16,17]. Further prediction and measurement of transitions confirmed the initial assignments. All the fits were completed using Pickett's SPFIT[14], using the same Hamiltonian as for the complexes with one and two water molecules. From the comparison of the experimental and theoretical rotational constants of the observed isomers we can unambiguously assign them to **3w-1**, **3w-3** and **3w-4**. The observation of all the monosubstituted ^{18}O isotopologues arising from the water molecules of **3w-1** further confirmed its assignment and allowed determination of its water oxygen coordinates (see table S12). No ^{18}O isotopologues could be detected for **3w-3** and **3w-4** due to the lower intensity of their rotational spectra.

We searched for other 1,4-NQ-$(H_2O)_3$ isomers predicted to be at low energy in the spectrum. However, no other species could be identified. The non-observation of **3w-2** can be explained by the lower predicted values of its dipole moment components in comparison with those of **3w-1**, **3w-3** and **3w-4**.

References

(1) Grimme, S.; Bannwarth, C.; Shushkov, P. A Robust and Accurate Tight-Binding Quantum Chemical Method for Structures, Vibrational Frequencies, and Noncovalent Interactions of Large Molecular Systems Parametrized for All Spd-Block Elements (Z = 1-86). *J. Chem. Theory Comput.* **2017**, *13*, 1989–2009.

(2) Bannwarth, C.; Ehlert, S.; Grimme, S. GFN2-XTB - An Accurate and Broadly Parametrized Self-Consistent Tight-Binding Quantum Chemical Method with Multipole Electrostatics and Density-Dependent Dispersion Contributions. *J. Chem. Theory Comput.* **2019**, *15*, 1652–1671.

(3) Frisch, M. J.; Trucks, G. W.; Schlegel, H. B.; Scuseria, G. E.; Robb, M. A.; Cheeseman, J. R.; Scalmani, G.; Barone, V.; Mennucci, B.; Petersson, G. A.; Nakatsuji, H.; Caricato, M.; Li, X.; Hratchian, H. P.; Izmaylov, A. F.; Bloino, J.; Zheng, G.; Sonnenberg, J. L.; Hada, M.; Ehara, M.; Toyota, K.; Fukuda, R.; Hasegawa, J.; Ishida, M.; Nakajima, T.; Honda, Y.; Kitao, O.; Nakai, H.; Vreven, T.; Montgomery Jr., J. A.; Peralta, J. E.; Ogliaro, F.; Bearpark, M.; Heyd, J. J.; Brothers, E.; Kudin, K. N.; Staroverov, V. N.; Kobayashi, R.; Normand, J.; Raghavachari, K.; Rendell, A.; Burant, J. C.; Iyengar, S. S.; Tomasi, J.; Cossi, M.; Rega, N.; Millam, J. M.; Klene, M.; Knox, J. E.; Cross, J. B.; Bakken, V.; Adamo, C.; Jaramillo, J.; Gomperts, R.; Stratmann, R. E.; Yazyev, O.; Austin, A. J.; Cammi, R.; Pomelli, C.; Ochterski, J. W.; Martin, R. L.; Morokuma, K.; Zakrzewski, V. G.; Voth, G. A.; Salvador, P.; Dannenberg, J. J.; Dapprich, S.; Daniels, A. D.; Farkas, Ö.; Foresman, J. B.; Ortiz, J. V; Cioslowski, J.; Fox, D. J. Gaussian09 Revision E.01, Gaussian Inc. Wallingford CT. *Gaussian 09 Revision E.01*. 2010.

(4) Boys, S. F.; Bernardi, F. The Calculation of Small Molecular Interactions by the Differences of Separate Total Energies. Some Procedures with Reduced Errors. *Mol. Phys.* **1970**, *19*, 553–566.

(5) Xantheas, S. S. On the Importance of the Fragment Relaxation Energy Terms in the Estimation of the Basis Set Superposition Error Correction to the Intermolecular Interaction Energy. *J. Chem. Phys.* **1996**, *104*, 8821–8824.

(6) Neese, F. The ORCA Program System. *WIREs Comput. Mol. Sci.* **2012**, *2*, 73–78.

(7) Neese, F. Software Update : The ORCA Program System, Version 4 . 0. *WIREs Comput. Mol. Sci.* **2018**, *8*, 4–9.

(8) Werner, H.-J.; Adler, T. B.; Manby, F. R. General Orbital Invariant MP2-F12 Theory. *J. Chem. Phys.* **2009**, *164102*.

(9) Johnson, E. R.; Keinan, S.; Mori-Sánchez, P.; Contreras-García, J.; Cohen, A. J.; Yang, W. Revealing Noncovalent Interactions. *J. Am. Chem. Soc.* **2010**, *132*, 6498–6506.

(10) Lu, T.; Chen, F. Multiwfn: A Multifunctional Wavefunction Analyzer. *J. Comput. Chem.* **2012**, *33*, 580–592.

(11) Loru, D.; Bermúdez, M. A.; Sanz, M. E. Structure of Fenchone by Broadband Rotational Spectroscopy. *J. Chem. Phys.* **2016**, *145*, 074311.

(12) Loru, D.; Quesada-Moreno, M. M.; Avilés-Moreno, J. R.; Jarman, N.; Huet, T. R.; López-González, J. J.; Sanz, M. E. Conformational Flexibility of Limonene Oxide Studied By Microwave Spectroscopy. *ChemPhysChem* **2017**, *18*, 268.

(13) Neill, J. L.; Shipman, S. T.; Alvarez-Valtierra, L.; Lesarri, A.; Kisiel, Z.; Pate, B. H. Rotational Spectroscopy of Iodobenzene and Iodobenzene-Neon with a Direct Digital 2-8 GHz Chirped-Pulse Fourier Transform Microwave Spectrometer. *J. Mol. Spectrosc.* **2011**, *269*, 21–29.

(14) Pickett, H. M. The Fitting and Prediction of Vibration-Rotation Spectra with Spin Interactions. *J. Mol. Spectrosc.* **1991**, *148*, 371–377.

(15) Watson, J. K. G. *Vibrational Spectra and Structure*, Vol. 6.; Elsevier: New York, 1977.

(16) Western, C. M. PGOPHER: A Program for Simulating Rotational, Vibrational and Electronic Spectra. *J. Quant. Spectrosc. Radiat. Transf.* **2017**, *186*, 221–242.

(17) Western, C. M.; Billinghurst, B. E. Automatic and Semi-Automatic Assignment and Fitting of Spectra with PGOPHER. *Phys. Chem. Chem. Phys.* **2019**, *21*, 13986–13999.

5. 1,4-Naphthoquinone-H_2O

Figure S2. Optimised structures of 1,4-NQ-H_2O isomers within 1000 cm^{-1}.

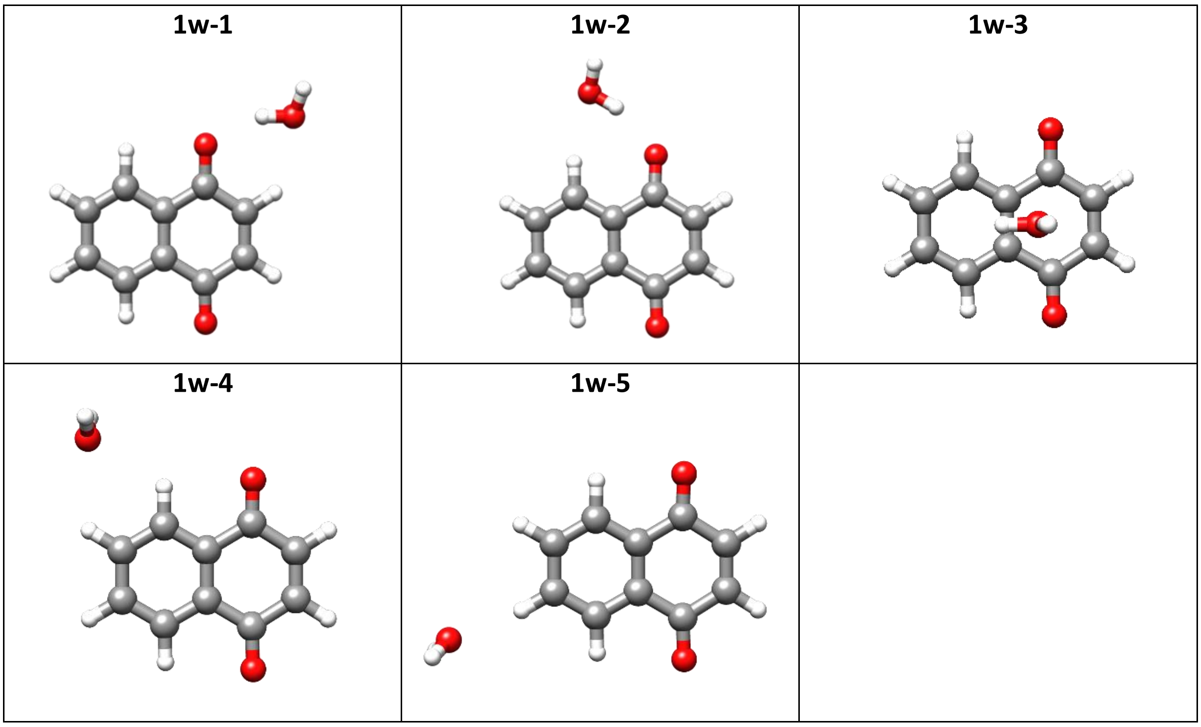

Table S1. MP2/6-311++G(d,p) spectroscopic parameters for 1,4-NQ-H_2O complexes.

Parameter	1w-1	1w-2	1w-3	1w-4	1w-5
A^a (MHz)	1203.5	1094.4	978.6	1191.2	1277.8
B (MHz)	585.6	657.9	837.4	564.6	468.0
C (MHz)	394.4	411.2	592.2	383.8	343.2
P_c (uÅ^2)	0.8	0.5	133.3	1.3	1.4
$\|\mu_a\|/\|\mu_b\|/\|\mu_c\|^b$ (D)	1.5/2.4/0.8	0.0/0.3/0.8	2.1/0.0/1.8	3.4/0.4/0.0	3.8/0.7/0.0
ΔE^c (cm^{-1})	0.0	86.8	540.8	1063.6	1156.8
ΔE_0^d (cm^{-1})	0.0	111.7	231.5	807.9	899.2
D_e^e(kJ mol^{-1})	-23.0	-27.2	-11.1	-10.8	-10.2

[a] *A*, *B*, *C* are the rotational constants. [b] $|\mu_a|$, $|\mu_b|$, $|\mu_c|$ are the absolute values of the electric dipole moment components along the principal inertial axes *a*, *b* and *c*. [c] ΔE are the energies relative to the lowest energy isomer. [d] ΔE_0 are the zero-point corrected energies. [e] Interaction energies energies including BSSE and fragment relaxation.

Table S2. B3LYP-D3BJ/6-311++G(d,p) spectroscopic parameters for 1,4-NQ-H_2O complexes.

Parameter	1w-1	1w-2	1w-3	1w-4	1w-5
A^a (MHz)	1212.5	1098.8	976.9	1197.6	1294.3
B (MHz)	593.0	670.7	831.4	568.7	471.1
C (MHz)	398.3	416.6	593.8	386.3	345.9
P_c (uÅ^2)	0.1	0.2	137.1	1.2	1.1
$\|\mu_a\|/\|\mu_b\|/\|\mu_c\|^b$ (D)	1.8/2.6/0.5	0.0/0.1/0.7	2.0/0.0/2.1	3.3/0.5/0.0	4.1/0.4/0.0
ΔE^c (cm^{-1})	0.0	101.3	1360.6	1474.2	1544.4
ΔE_0^d (cm^{-1})	0.0	103.4	1029.1	1181.7	1220.5
D_e^e(kJ mol^{-1})	-29.7	-28.3	-12.2	-11.5	-10.7

[a] *A*, *B*, *C* are the rotational constants. [b] $|\mu_a|$, $|\mu_b|$, $|\mu_c|$ are the absolute values of the electric dipole moment components along the principal inertial axes *a*, *b* and *c*. [c] ΔE are the energies relative to the lowest energy isomer. [d] ΔE_0 are the zero-point corrected energies. [e] Interaction energies energies including BSSE and fragment relaxation.

Table S3. Experimental spectroscopic parameters of the $H_2{}^{18}O$ isotopologue of isomer **1w-1** of 1,4-NQ-H_2O.

**Parameter**	**^{18}O**
A^a (MHz)	1209.40227(76)^f
B (MHz)	555.91184(48)
C (MHz)	381.16213(32)
$P_c{}^b$(uÅ^2)	0.54340(28)
Δ_J (kHz)	0.0262(35)
Δ_{JK} (kHz)	0.111(24)
Δ_K (kHz)	[-0.0382]^g
δ_J (kHz)	0.0101(20)
δ_K (kHz)	[0.089]
μ_a/μ_b /$\mu_c{}^c$ (D)	y/y/n
N^d	50
σ^e (kHz)	6.7

[a] A, B and C are the rotational constants, Δ_J, Δ_{JK}, δ_J and δ_K are the quartic centrifugal distortion constants. [b] Planar moment of inertia $P_c = \sum_i m_i c_i^2$. [c] Yes (y) or no (n) observation of μ_a-, μ_b- and μ_c-type transitions. [d] Number of rotational transitions included in the fit. [e] Rms deviation of the fit. [f] Standard error in parentheses in the units of the last digit. [g] Parameters in square brackets were fixed to the values of the parent species.

6. 1,4-Naphthoquinone-$(H_2O)_2$

Figure S3. Optimised structures of 1,4-NQ-$(H_2O)_2$ isomers within 1000 cm^{-1}.

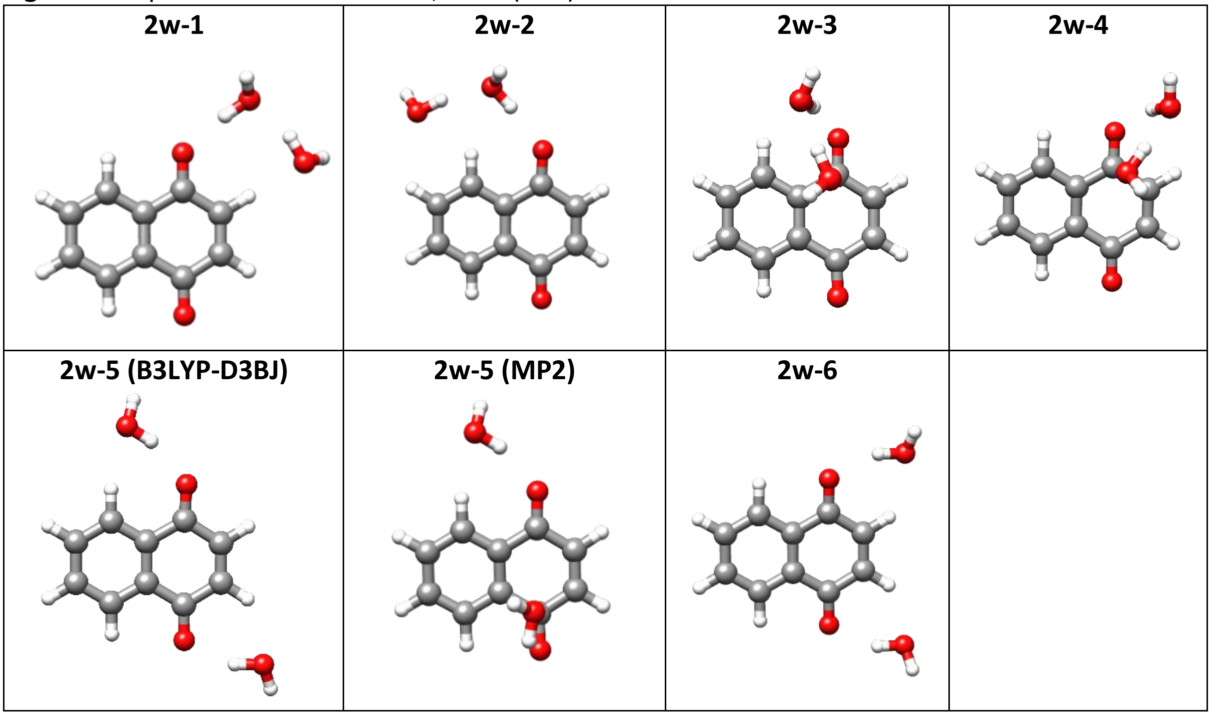

Table S4. MP2/6-311++G(d,p) spectroscopic parameters for 1,4-NQ-$(H_2O)_2$ complexes.

Parameter	2w-1	2w-2	2w-3	2w-4	2w-5	2w-6
A^a (MHz)	1037.1	966.5	817.8	917.9	989.6	594.4
B (MHz)	391.5	450.1	609.6	523.5	410.7	576.6
C (MHz)	284.6	308.0	434.3	394.3	290.6	293.1
P_c (uÅ^2)	1.2	2.4	141.7	117.1	1.1	1.2
$\|\mu_a\|/\|\mu_b\|/\|\mu_c\|^b$ (D)	1.4/2.9/0.1	0.4/1.3/0.2	1.6/1.7/0.5	1.1/1.5/0.1	0.1/1.3/1.6	4.0/0.1/1.6
ΔE^c (cm^{-1})	0.0	164.1	222.8	226.3	1102.1	1165.6
$\Delta E_0{}^d$ (cm^{-1})	0.0	125.8	344.1	410.9	1192.0	1204.9
$D_e{}^e$(kJ mol^{-1})	-52.6	-50.7	-44.8	-45.0	-43.0	-42.9

[a] A, B, C are the rotational constants. [b] $|\mu_a|$, $|\mu_b|$, $|\mu_c|$ are the absolute values of the electric dipole moment components along the principal inertial axes a, b and c. [c] ΔE are the energies relative to the lowest energy isomer. [d] ΔE_0 are the zero-point corrected energies. [e] Interaction energies energies including BSSE and fragment relaxation.

Table S5. B3LYP-D3BJ/6-311++G(d,p) spectroscopic parameters for 1,4-NQ-$(H_2O)_2$ complexes.

Parameter	2w-1	2w-2	2w-3	2w-4	2w-5	2-6
A^a (MHz)	1046.0	972.3	827.3	933.1	995.3	602.6
B (MHz)	398.6	455.3	604.6	507.9	419.1	584.8
C (MHz)	288.9	310.8	432.0	385.6	295.0	296.9
P_c (uÅ^2)	0.9	1.9	138.5	113.0	0.2	0.3
$\|\mu_a\|/\|\mu_b\|/\|\mu_c\|^b$ (D)	1.7/3.0/0.1	0.2/1.1/0.1	1.2/1.9/0.7	0.6/1.8/0.3	0.1/1.6/1.2	4.4/0.0/0.0
ΔE^c (cm^{-1})	0.0	313.0	675.9	708.9	1099.6	1109.8
$\Delta E_0{}^d$ (cm^{-1})	0.0	212.5	515.1	583.1	829.4	856.6
$D_e{}^e$(kJ mol^{-1})	-68.6	-65.4	-60.0	-60.2	-58.1	-58.1

[a] A, B, C are the rotational constants. [b] $|\mu_a|$, $|\mu_b|$, $|\mu_c|$ are the absolute values of the electric dipole moment components along the principal inertial axes a, b and c. [c] ΔE are the energies relative to the lowest energy isomer. [d] ΔE_0 are the zero-point corrected energies. [e] Interaction energies energies including BSSE and fragment relaxation.

Table S6. Experimental spectroscopic parameters for the $H_2{}^{18}O$ isotopologues of 1,4-NQ-$(H_2O)_2$ complexes.

		2w-1		2w-2	
Parameter	16-18	18-16	18-18	16-18	18-16
A^a (MHz)	1036.85718(64)[f]	1032.08472(58)	1025.57191(52)	955.96796(66)	960.55449(70)
B (MHz)	379.29866(17)	380.77578(15)	369.69550(14)	437.49167(22)	434.42877(32)
C (MHz)	278.04323(19)	278.48984(16)	272.06191(13)	300.65941(17)	299.65448(22)
$P_c{}^b$(uÅ^2)	1.0934(11)	1.0972(15)	1.1019(5)	1.4418(17)	1.4340(10)
Δ_J (kHz)	0.00514(89)	0.00607(76)	0.00565(42)	0.00772(82)	0.0085(10)
Δ_{JK} (kHz)	0.268(18)	0.273(17)	0.234(14)	0.218(16)	0.226(17)
Δ_K (kHz)	[-0.145][g]	[-0.145]	[-0.145]	[-0.128]	[-0.128]
δ_K (kHz)	[0.084]	[0.084]	[0.084]	[0.0553]	[0.0553]
μ_a/μ_b /$\mu_c{}^c$ (D)	n/y/n	n/y/n	n/y/n	n/y/n	n/y/n
N[d]	42	41	33	35	37
σ[e] (kHz)	5.2	4.4	3.5	4.4	5.8

[a] A, B and C are the rotational constants, Δ_J, and Δ_{JK}, are the quartic centrifugal distortion constants. [b] Planar moment of inertia $P_c = \sum_i m_i c_i^2$. [c] Yes (y) or no (n) observation of μ_a-, μ_b- and μ_c-type transitions. [d] Number of rotational transitions included in the fit. [e] Rms deviation of the fit. [f] Standard error in parentheses in the units of the last digit. [g] Parameters in square brackets were fixed to the values of the parent species.

.

7. 1,4-Naphthoquinone-$(H_2O)_3$

Figure S4. Optimised structures of 1,4-NQ-$(H_2O)_3$ isomers within 1000 cm^{-1}.

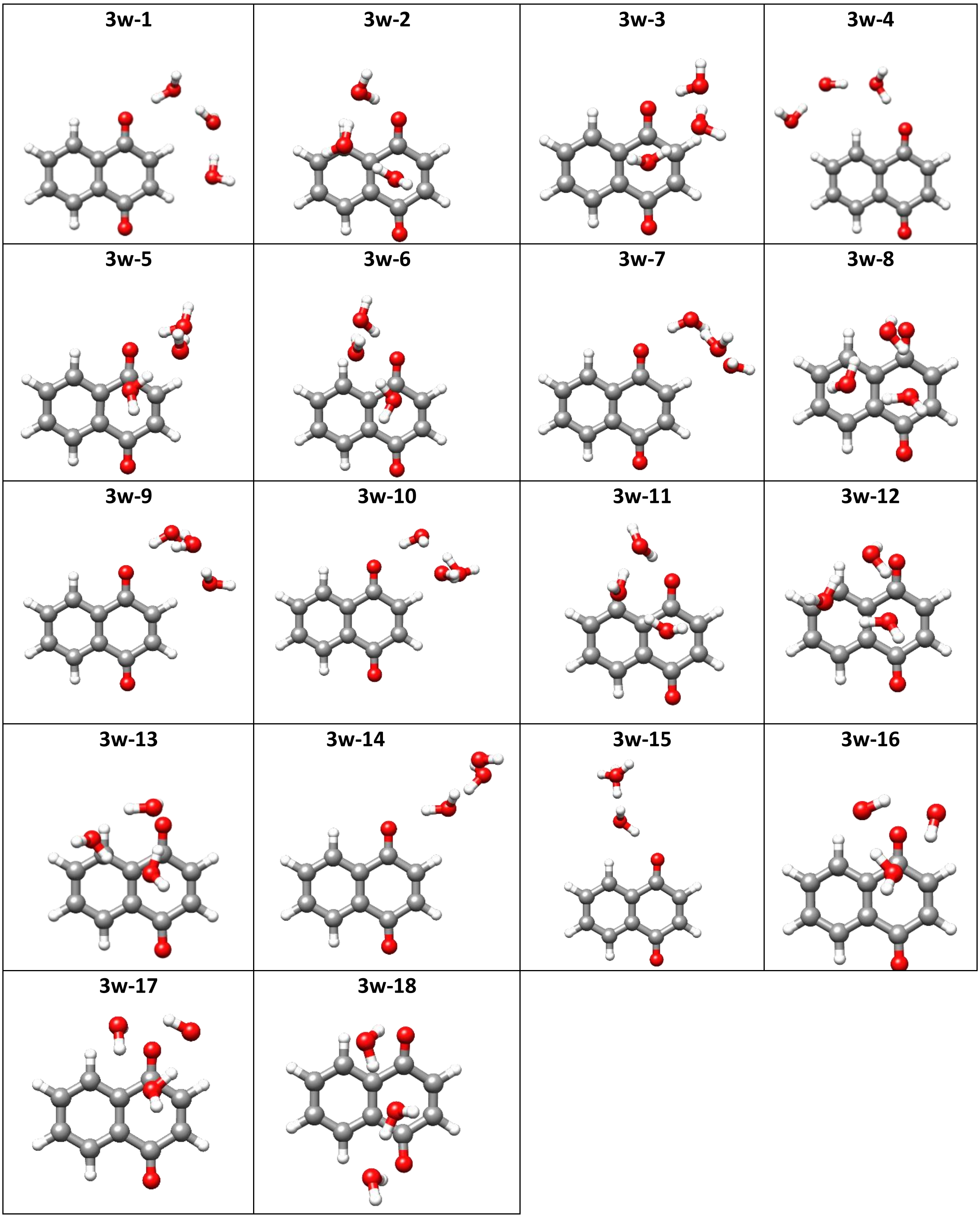

Figure S5. Different optimised structures of 1,4-NQ-$(H_2O)_3$ at MP2 and B3LYP-D3BJ level of theories.

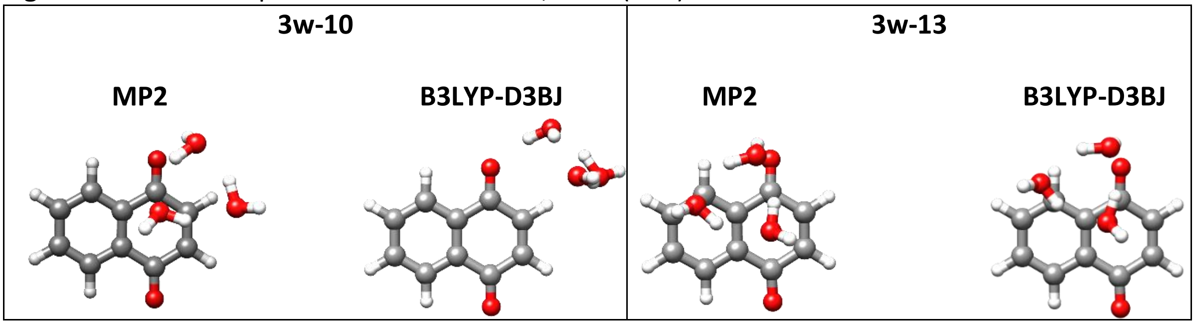

Table S7. MP2/6-311++G(d,p) spectroscopic parameters for 1,4-NQ-$(H_2O)_3$ complexes.

**Parameter**	**3w-1**	**3w-2**	**3w-3**	**3w-4**	**3w-5**	**3w-6**	**3w-7**	**3w-8**	**3w-9**
A^a (MHz)	825.7	680.4	804.2	764.8	812.3	720.1	895.6	608.7	917.8
B (MHz)	315.9	528.5	400.8	325.9	368.2	448.0	291.1	572.6	275.5
C (MHz)	230.5	425.3	331.4	231.9	314.4	359.7	236.6	468.1	224.1
P_c (uÅ^2)	9.7	255.4	182.2	16.1	193.6	212.4	82.2	316.6	64.9
$\|\mu_a\|^b$ (D)	1.2	1.8	1.0	0.1	1.4	2.6	0.5	0.7	2.4
$\|\mu_b\|$	3.4	0.1	2.2	2.5	2.3	0.7	0.2	1.4	0.0
$\|\mu_c\|$	0.6	1.1	0.1	0.7	0.5	0.3	0.4	1.6	0.7
ΔE^c (cm^{-1})	761.4	0.0	338.6	928.9	703.2	716.7	968.5	211.4	1068.4
ΔE_0^d (cm^{-1})	831.4	0.0	410.4	1047.6	748.8	1047.6	1211.9	233.7	1276.5
D_e^e(kJ mol^{-1})	-82.8	-78.8	-79.7	-81.1	-76.9	-76.2	-80.9	-68.6	-80.4
**Parameter**	**3w-10**	**3w-11**	**3w-12**	**3w-13**	**3w-14**	**3w-15**	**3w-16**	**tb27**	**3w-18**
A^a (MHz)	786.8	684.6	614.1	592.0	Not	942.4	608.7	641.4	613.6
B (MHz)	423.4	506.8	563.8	572.5	Conver-	227.7	572.6	463.9	517.0
C (MHz)	336.4	405.5	462.2	479.5	ged	193.6	468.1	388.8	454.4
P_c (uÅ^2)	166.8	244.5	313.0	341.2		72.7	316.6	288.8	344.5
$\mu_a{}^b$ (D)	0.8	3.4	0.8	0.6		0.5	0.7	1.0	4.5
μ_b	0.3	0.5	1.0	1.5		0.3	1.4	0.1	0.5
μ_c	1.4	0.6	0.7	0.7		0.2	1.6	0.6	1.4
ΔE^c (cm^{-1})	696.8	651.7	461.1	570.5		1490.7	211.4	842.7	1059.4
ΔE_0^d (cm^{-1})	722.7	534.4	414.4	515.1		1726.4	233.7	875.3	933.9
D_e^e(kJ mol^{-1})	-77.3	-73.8	-75.0	-73.3		-77.6	-68.6	-74.9	-68.2

[a] A, B, C are the rotational constants. [b] $|\mu_a|$, $|\mu_b|$, $|\mu_c|$ are the absolute values of the electric dipole moment components along the principal inertial axes a, b and c. [c] ΔE are the energies relative to the lowest energy isomer. [d] ΔE_0 are the zero-point corrected energies. [e] Interaction energies energies including BSSE and fragment relaxation.

Table S8. B3LYP-D3BJ/6-311++G(d,p) spectroscopic parameters for 1,4-NQ-$(H_2O)_3$ complexes.

Parameter	3w-1	3w-2	3w-3	3w-4	3w-5	3w-6	3w-7	3w-8	3w-9
A^a (MHz)	836.7	699.6	816.1	778.3	821.9	732.9	908.1	613.5	927.1
B (MHz)	318.5	501.3	389.0	330.5	367.0	433.5	290.6	570.8	278.5
C (MHz)	233.0	400.0	326.6	234.9	313.6	350.3	235.6	459.5	225.4
P_c (uÅ^2)	10.9	233.5	185.5	13.5	190.2	206.3	75.2	304.7	58.8
$\|\mu_a\|^b$ (D)	1.6	1.7	0.8	0.1	1.0	2.5	0.8	1.2	2.7
$\|\mu_b\|$	3.3	0.4	2.5	2.2	2.6	1.0	0.4	1.5	0.3
$\|\mu_c\|$	0.5	1.3	0.4	0.6	0.7	0.9	0.0	1.5	0.9
ΔE^c (cm^{-1})	0.0	14.9	76.3	173.9	323.1	332.0	126.4	191.1	212.0
ΔE_0^d (cm^{-1})	0.0	91.7	117.9	174.7	355.5	363.0	370.5	409.1	443.6
D_e^e(kJ mol^{-1})	-107.9	-105.4	-105.4	-105.5	-102.3	-101.8	-106.5	-103.2	-105.4

Parameter	3w-10	3w-11	3w-12	3w-13	3w-14	3w-15	3w-16	3w-17	3w-18
A^a (MHz)	899.0	718.3	641.2	685.1	1021.7	947.5	757.0	745.4	618.5
B (MHz)	302.5	469.3	537.4	488.7	211.1	232.3	446.7	454.4	516.4
C (MHz)	246.3	373.4	423.7	370.0	183.4	196.9	337.7	341.8	454.8
P_c (uÅ^2)	90.5	213.5	267.9	203.0	66.5	71.1	304.7	155.8	342.3
$\|\mu_a\|^b$ (D)	0.8	3.3	0.4	0.2	2.3	0.8	3.1	2.6	4.2
$\|\mu_b\|$	0.2	0.5	1.3	1.6	2.0	0.5	1.3	0.3	0.5
$\|\mu_c\|$	0.3	1.0	0.8	1.1	0.4	0.1	0.4	0.6	1.8
ΔE^c (cm^{-1})	216.2	565.2	465.7	608.3	621.6	702.8	826.9	855.0	1147.3
ΔE_0^d (cm^{-1})	449.7	514.9	595.4	720.1	858.8	934.5	996.9	1018.8	1114.7
D_e^e(kJ mol^{-1})	-105.6	-99.6	-100.0	-99.7	-102.1	-101.0	-96.8	-92.1	-105.6

[a] *A*, *B*, *C* are the rotational constants. [b] $|\mu_a|$, $|\mu_b|$, $|\mu_c|$ are the absolute values of the electric dipole moment components along the principal inertial axes *a*, *b* and *c*. [c] ΔE are the energies relative to the lowest energy isomer. [d] ΔE_0 are the zero-point corrected energies. [e] Interaction energies energies including BSSE and fragment relaxation.

Table S9. B3LYP-D4/def2-TZVP spectroscopic parameters for 1,4-NQ-$(H_2O)_3$ complexes.

Parameter	3w-1	3w-2	3w-3	3w-4	3w-5	3w-6	3w-7	3w-8	3w-9
A^a (MHz)	840.8	700.4	817.8	779.2	823.0	733.1	913.3	614.9	923.3
B (MHz)	317.9	498.6	387.3	329.7	367.4	434.3	290.1	570.1	282.6
C (MHz)	233.3	399.3	326.4	234.7	314.3	350.8	235.1	457.7	229.1
$\|\mu_a\|^b$ (D)	1.5	1.7	0.8	0.0	1.0	2.5	0.6	1.3	2.7
$\|\mu_b\|$	3.1	0.3	2.5	2.1	2.5	0.8	0.3	1.4	0.1
$\|\mu_c\|$	0.4	1.3	0.4	0.8	0.7	0.9	0.1	1.3	0.8
ΔE^c (cm^{-1})	194.8	84.8	79.9	455.8	302.4	389.0	95.1	0.0	183.4
ΔE_0^d (cm^{-1})	137.4	0.0	48.6	352.5	186.5	259.9	311.9	196.2	390.5

Parameter	3w-10	3w-11	3w-12	3w-13	3w-14	3w-15	3w-16	3w-17	3w-18
A^a (MHz)	894.4	724.0	632.5	703.1	1020.4	948.0	762.8	742.1	621.4
B (MHz)	303.4	465.1	547.3	476.6	212.1	232.4	440.5	459.2	517.4
C (MHz)	247.6	370.6	435.2	358.3	184.1	197.0	333.9	345.8	455.2
$\|\mu_a\|^b$ (D)	0.6	3.3	0.5	0.3	2.3	0.9	3.0	2.4	4.1
$\|\mu_b\|$	0.2	0.5	1.2	1.7	1.9	0.4	1.2	0.2	0.7
$\|\mu_c\|$	0.5	1.0	1.0	1.4	0.4	0.0	0.2	0.6	1.8
ΔE^c (cm^{-1})	160.7	585.1	274.4	370.8	554.7	647.2	507.1	519.2	1071.3
ΔE_0^d (cm^{-1})	359.6	434.2	374.1	484.8	767.6	888.9	672.0	656.7	1005.1

[a] *A*, *B*, *C* are the rotational constants. [b] $|\mu_a|$, $|\mu_b|$, $|\mu_c|$ are the absolute values of the electric dipole moment components along the principal inertial axes *a*, *b* and *c*. [c] ΔE are the energies relative to the lowest energy isomer. [d] ΔE_0 are the zero-point corrected energies.

Table S10. WB97X-D3/def2-TZVP spectroscopic parameters for 1,4-NQ-$(H_2O)_3$ complexes.

Parameter	3w-1	3w-2	3w-3	Xtb47	3w-5	3w-6	3w-7	3w-8	3w-9
A^a (MHz)	819.9	682.4	812.0	742.3	838.3	704.1	873.0	594.3	940.6
B (MHz)	324.1	528.4	364.6	355.7	320.1	449.3	274.2	574.1	217.7
C (MHz)	236.2	414.6	313.6	244.1	270.9	350.1	228.4	468.3	183.1
$\|\mu_a\|^b$ (D)	1.2	1.4	0.7	0.1	0.1	2.8	0.7	1.8	3.1
$\|\mu_b\|$	3.5	0.1	2.2	2.5	3.3	1.4	0.5	1.4	0.2
$\|\mu_c\|$	0.1	1.5	1.1	0.2	0.9	0.5	0.2	1.6	0.6
ΔE^c (cm^{-1})	483.3	236.8	525.5	965.8	900.7	984.3	550.9	0.0	1274.9
ΔE_0^d (cm^{-1})	310.3	138.0	431.5	602.7	837.6	820.3	622.0	0.0	1262.4
**Parameter**	**3w-10**	**3w-11**	**3w-12**	**3w-13**	**3w-14**	**3w-15**	**3w-16**	**3w-17**	**3w-18**
A^a (MHz)	867.0	740.5	593.3	636.9	987.2	910.0	760.2	751.2	614.1
B (MHz)	210.1	435.2	541.9	547.9	204.0	244.3	447.4	447.1	510.9
C (MHz)	182.8	354.4	463.6	431.6	179.4	206.8	341.5	337.5	451.3
$\|\mu_a\|^b$ (D)	1.8	2.4	0.8	.8	2.3	1.4	3.1	2.7	4.2
$\|\mu_b\|$	1.3	1.0	1.0	-1.3	1.9	0.1	1.1	0.2	1.6
$\|\mu_c\|$	1.6	1.2	1.3	1.2	0.2	0.4	0.7	0.8	2.1
ΔE^c (cm^{-1})	2450.4	1207.2	715.2	730.2	1055.9	1265.0	516.9	615.7	1425.2
ΔE_0^d (cm^{-1})	2010.5	899.3	507.9	635.9	1049.8	1183.2	564.7	624.8	1119.6

[a] A, B, C are the rotational constants. [b] $|\mu_a|$, $|\mu_b|$, $|\mu_c|$ are the absolute values of the electric dipole moment components along the principal inertial axes a, b and c. [c] ΔE are the energies relative to the lowest energy isomer. [d] ΔE_0 are the zero-point corrected energies.

Table S11. B2PLYP-D3BJ/def2-TZVP spectroscopic parameters for 1,4-NQ-$(H_2O)_3$ complexes.

Parameter	3w-1	3w-2	3w-3	3w-4	3w-5	3w-6	3w-7	3w-8	3w-9
A^a (MHz)	837.5	700.2	818.0	777.7	820.2	732.7	910.0	612.6	926.4
B (MHz)	318.9	502.0	388.1	329.1	366.1	432.3	291.0	573.5	280.1
C (MHz)	233.7	401.1	326.9	234.3	313.3	349.5	236.2	463.3	227.1
$\|\mu_a\|^b$ (D)	1.5	1.7	0.9	0.0	1.0	2.5	0.5	1.1	2.6
$\|\mu_b\|$	3.3	0.3	2.4	2.3	2.5	0.8	0.3	1.4	0.1
$\|\mu_c\|$	0.6	1.2	0.4	0.8	1.1	0.9	0.1	1.4	0.9
ΔE^c (cm^{-1})	241.0	204.9	253.0	507.7	466.8	528.0	172.8	0.0	237.2
ΔE_0^d (cm^{-1})	35.2	5.9	49.0	249.7	280.4	308.9	268.2	0.0	323.5
**Parameter**	**3w-10**	**3w-11**	**3w-12**	**3w-13**	**3w-14**	**3w-15**	**3w-16**	**3w-17**	**3w-18**
A^a (MHz)	900.5	728.6	630.3	711.9	1022.2	949.2	758.5	745.0	624.1
B (MHz)	302.3	460.7	550.9	470.1	211.3	231.3	447.7	458.2	518.4
C (MHz)	246.6	366.4	439.9	352.1	183.8	196.2	338.4	344.3	454.3
μ_a^b (D)	0.6	3.3	0.5	0.2	2.1	0.8	2.9	2.4	4.2
μ_b	0.1	0.5	1.2	1.6	2.0	0.4	1.2	0.2	0.8
μ_c	0.4	1.1	0.9	1.5	0.3	0.1	0.3	0.6	1.8
ΔE^c (cm^{-1})	239.5	766.0	384.8	489.3	594.4	701.5	597.0	615.7	1150.4
ΔE_0^d (cm^{-1})	292.5	447.5	281.7	404.1	699.6	813.1	585.3	569.1	897.7

[a] A, B, C are the rotational constants. [b] $|\mu_a|$, $|\mu_b|$, $|\mu_c|$ are the absolute values of the electric dipole moment components along the principal inertial axes a, b and c. [c] ΔE are the energies relative to the lowest energy isomer. [d] ΔE_0 are the zero-point corrected energies.

Table S12. Experimental spectroscopic parameters for the $H_2^{18}O$ isotopologues of the isomer **3w-1** of 1,4-NQ-$(H_2O)_3$.

Parameter	16-16-18	16-18-16	18-16-16	16-18-18	18-16-18	18-18-16
A^a (MHz)	814.57156(30)[f]	826.54552(46)	812.41914(38)	813.29335(68)	798.82552(71)	811.30443(81)
B (MHz)	309.268536(99)	305.57891(16)	309.80908(12)	300.92984(20)	305.08506(22)	301.42267(24)
C (MHz)	227.297405(69)	226.33733(11)	227.397481(83)	222.83307(14)	223.82224(17)	222.92897(18)
P_c^b (uÅ^2)	15.55773(84)	16.21350(93)	15.44336(66)	16.41266(88)	15.6161(13)	16.2888(11)
Δ_J (kHz)	0.02423(20)	0.02574(31)	0.02410(24)	0.02555(44)	0.02279(53)	0.02563(56)
Δ_{JK} (kHz)	[-0.0616][g]	[-0.0616]	[-0.0616]	[-0.0616]	[-0.0616]	[-0.0616]
Δ_K (kHz)	0.283(15)	0.322(23)	0.284(18)	0.318(33)	0.306(38)	0.324(37)
δ_J (kHz)	[0.00586]	[0.00586]	[0.00586]	[0.00586]	[0.00586]	[0.00586]
$\mu_a/\mu_b/\mu_c^c$ (D)	n/y/n	n/y/n	n/y/n	n/y/n	n/y/n	n/y/n
N[d]	54	53	52	44	40	37
σ[e] (kHz)	2.6	3.9	3.1	4.9	5.3	5.3

[a]*A*, *B* and *C* are the rotational constants, Δ_J, and Δ_K, are the quartic centrifugal distortion constants. [b] Planar moment of inertia $P_c = \sum_i m_i c_i^2$. [c] Yes (y) or no (n) observation of μ_a-, μ_b- and μ_c-type transitions. [d] Number of rotational transitions included in the fit. [e] Rms deviation of the fit. [f]Standard error in parentheses in the units of the last digit. [g] Parameters in square brackets were fixed to the values of the parent species.

Table S13. Comparison of experimental and theoretical rotational constants for the observed 1,4-NQ-$(H_2O)_3$ complexes $(A_{calc} - A_{exp})/A_{exp} \times 100\%$.

		Exp.	B3LYP-D3BJ	%	MP2	%	B3LYP-D4	%	wB97X-D3	%	B2PLYP-D3BJ	%	RI-MP2-aug	%
	A	827.7359	836.7	1.1	825.7	-0.2	840.8	1.6	828.1	0.0	837.5	1.2	836.4	1.0
**3w-1**	*B*	314.3125	318.5	1.3	315.9	0.5	317.9	1.1	324.0	3.1	318.9	1.5	319.0	1.5
	C	231.0118	233.0	0.9	230.5	-0.2	233.3	1.0	236.7	2.5	233.7	1.2	233.9	1.3
	av[a]			1.1		0.3		1.2		1.9		1.3		1.3
	A	807.7829	816.1	1.0	804.2	-0.4	817.8	1.2	812.0	0.5	818.0	1.3	820.7	1.6
**3w-3**	*B*	381.6797	389.0	1.9	400.8	5.0	387.3	1.5	364.6	-4.5	388.1	1.7	399.2	4.6
	C	320.7252	326.6	1.8	331.4	3.3	326.4	1.8	313.6	-2.2	326.9	1.9	331.2	3.3
	av[a]			1.6		2.9		1.5		2.4		1.6		3.2
	A	763.9906	778.3	1.9	764.8	0.1	779.2	2.0	716.7	-6.2	777.7	1.8	773.8	1.3
**3w-4**	*B*	328.8964	330.5	0.5	325.9	-0.9	329.7	0.2	342.2	4.0	329.1	0.1	332.8	1.2
	C	234.6890	234.9	0.1	231.9	-1.2	234.7	0.0	234.6	0.0	234.3	-0.2	236.9	0.9
	av[a]			0.8		0.7		0.7		3.4		0.7		1.1

[a]Average deviation, considering the absolute values of the deviation for *A*, *B*, and *C* rotational constants.

8. 1,4-Naphthoquinone-$(H_2O)_4$

Figure S5. Optimised structures of 1,4-NQ-$(H_2O)_4$ isomers within 600 cm^{-1}.

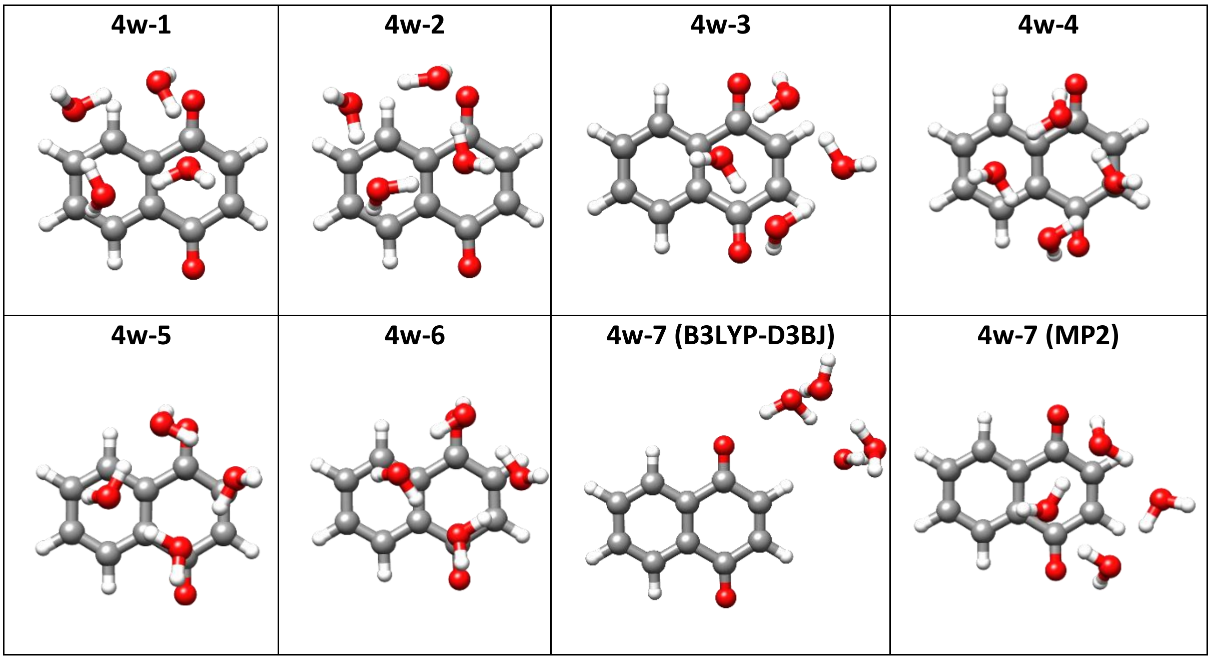

Table S14. MP2/6-311++G(d,p) spectroscopic parameters for the 1,4-NQ-$(H_2O)_4$ complexes.

Parameter	4w-1	4w-2	4w-3	4w-4	4w-5	4w-6	4w-7
A^a (MHz)	590.5	572.7	681.8	500.3	538.9	573.9	683.0
B (MHz)	438.2	446.5	384.3	476.9	473.9	433.3	383.7
C (MHz)	351.1	361.8	311.1	443.3	397.3	368.0	310.4
$\|\mu_a\|^b$ (D)	0.2	0.0	1.9	0.8	1.1	1.3	1.9
$\|\mu_b\|$	0.8	0.9	0.0	0.7	0.5	0.5	0.0
$\|\mu_c\|$	0.7	0.8	0.0	0.9	0.4	0.5	0.0
ΔE^c (cm^{-1})	0.0	166.4	344.6	357.6	252.0	374.2	276.7
$\Delta E_0{}^d$ (cm^{-1})	0.0	125.8	160.0	111.1	182.8	330.5	212.2

[a] A, B, C are the rotational constants. [b] $|\mu_a|$, $|\mu_b|$, $|\mu_c|$ are the absolute values of the electric dipole moment components along the principal inertial axes a, b and c. [c] ΔE are the energies relative to the lowest energy isomer. [d] ΔE_0 are the zero-point corrected energies.

Table S15. B3LYP-D3BJ/6-311++G(d,p) spectroscopic parameters for the 1,4-NQ-$(H_2O)_4$ complexes.

Parameter	4w-1	4w-2	4w-3	4w-4	4w-5	4w-6	4w-7
A^a (MHz)	594.3	586.3	679.8	514.5	550.6	584.6	759.8
B (MHz)	431.3	434.8	385.8	459.8	460.4	430.3	214.2
C (MHz)	346.5	347.4	314.6	429.9	388.3	359.9	181.4
$\|\mu_a\|^b$ (D)	0.1	0.0	2.2	1.6	1.7	1.7	1.6
$\|\mu_b\|$	1.0	1.1	0.0	0.2	0.4	0.5	0.5
$\|\mu_c\|$	0.6	0.6	0.2	0.1	0.6	0.6	0.4
ΔE^c (cm^{-1})	0.0	135.0	266.0	416.0	424.7	443.7	538.3
$\Delta E_0{}^d$ (cm^{-1})	0.0	151.4	302.0	360.8	339.7	347.0	563.2

[a] A, B, C are the rotational constants. [b] $|\mu_a|$, $|\mu_b|$, $|\mu_c|$ are the absolute values of the electric dipole moment components along the principal inertial axes a, b and c. [c] ΔE are the energies relative to the lowest energy isomer. [d] ΔE_0 are the zero-point corrected energies.

9. Structural analysis

Table S16. Substitution (r_s) and B3LYP-D3BJ/6-311++G(d,p) coordinates in Å of the oxygen atoms of the water molecules in 1,4-NQ-$(H_2O)_{1-3}$ complexes.

		|**a**|		|**b**|		|**c**|	
		**Expt.**	**Theor.**	**Expt.**	**Theor.**	**Expt.**	**Theor.**
**1w-1**	O1[a]	4.6521(3)[b]	4.5869	0.3620(2)	-0.3686	0.0303(6)	-0.0479
**2w-1**	O1	4.2302(4)	4.2271	1.6117(9)	-1.5841	0.0500(3)	0.0166
	O2	4.5323(3)	4.4516	1.1915(3)	1.1706	0.0503(2)	-0.0366
**2w-2**	O1	3.7671(4)	3.7385	1.6457(9)	-1.6117	0.0768(7)	-0.1434
	O2	4.2827(4)	4.2470	1.1826(3)	1.1529	0.0415(5)	0.0731
**3w-1**	O1	3.4107(4)	3.3816	2.4316(6)	2.4166	0.178(9)	0.1390
	O2	4.7709(3)	4.7714	0.160(9)	0.1500	0.657(2)	-0.5420
	O3	3.6083(4)	3.5583	2.2399(7)	-2.2402	0.302(5)	0.2117

[a] Labelling of oxygens are according to Figure 1. [b] Errors include Costain's error.

Figure S7. Plots of the reduced density gradient (RDG) versus sign(λ_2)ρ for the observed isomers of 1,4-NQ-H_2O, 1,4-NQ-$(H_2O)_2$ and 1,4-NQ-$(H_2O)_3$.

1w-1

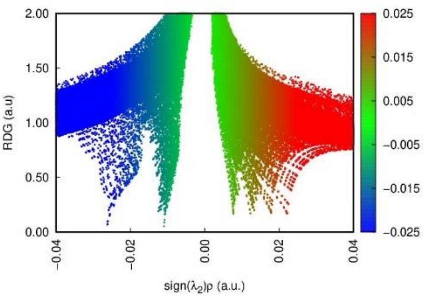

2w-1

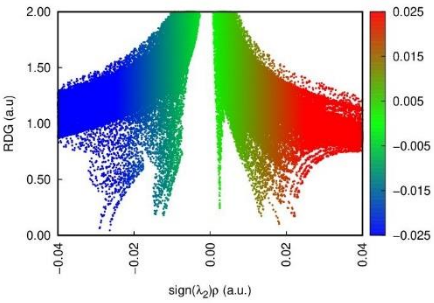

2w-2

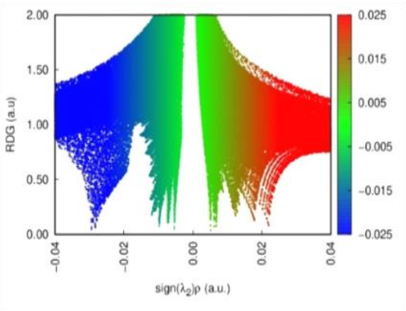

3w-1

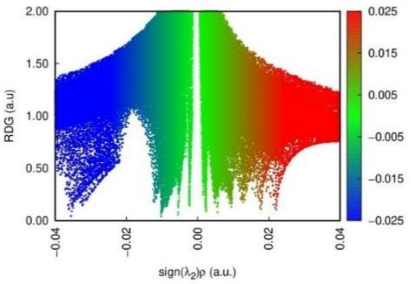

3w-3

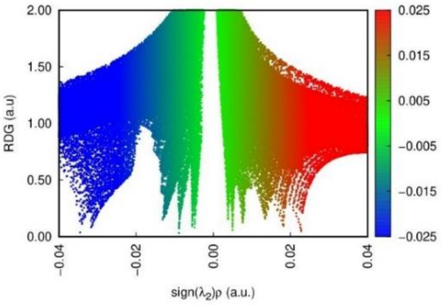

3w-4

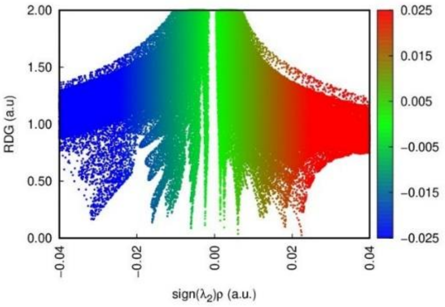

10. Tables of frequencies

Table S17. Measured frequencies and residuals (in MHz) for the rotational transitions of the isomer **1w-1** of 1,4-NQ-H_2O.

J’	K'_{-1}	K'_{+1}	J”	K''_{-1}	K''_{+1}	ν_{obs}	$\nu_{obs}-\nu_{calc}$
4	1	3	3	2	2	2578.4457	-0.0065
3	1	3	2	1	2	2626.9369	0.0002
8	3	5	8	2	6	2788.2486	-0.0011
6	3	3	6	2	4	2792.1550	-0.0025
3	0	3	2	0	2	2793.2636	-0.0004
5	1	4	5	0	5	2809.9830	-0.0064
3	2	2	2	2	1	2932.3074	0.0072
3	2	2	2	2	1	2932.3074	0.0072
5	3	2	5	2	3	3018.8205	0.0083
3	2	1	2	2	0	3071.3310	-0.0072
3	1	3	2	0	2	3100.9620	0.0002
9	3	6	9	2	7	3141.3130	0.0085
4	2	3	4	1	4	3165.6481	-0.0055
3	1	2	2	1	1	3190.7587	0.0035
4	3	1	4	2	2	3268.1535	-0.0025
4	0	4	3	1	3	3295.3190	0.0038
4	1	4	3	1	3	3468.8378	0.0015
4	0	4	3	0	3	3603.0144	0.0013
3	3	1	3	2	2	3628.0425	0.0042
5	2	4	5	1	5	3678.9914	-0.0077
6	1	5	6	0	6	3688.7972	0.0038
8	2	6	8	1	7	3747.8668	0.0011
9	4	5	9	3	6	3751.2391	-0.0007
10	3	7	10	2	8	3761.0147	-0.0052
4	1	4	3	0	3	3776.5346	0.0005
5	1	4	4	2	3	3848.6977	0.0029
4	2	3	3	2	2	3880.7974	-0.0026
5	3	3	5	2	4	3886.7248	0.0024
4	3	2	3	3	1	3971.6901	-0.0003
4	3	1	3	3	0	3998.4728	-0.0005
2	2	1	1	1	0	4024.2562	-0.0037
8	4	4	8	3	5	4074.5552	-0.0010
6	3	4	6	2	5	4149.9116	0.0020
4	2	2	3	2	1	4187.0493	0.0002
4	1	3	3	1	2	4199.9316	0.0108
5	0	5	4	1	4	4204.3614	0.0025
2	2	0	1	1	1	4250.5802	0.0010
6	2	5	6	1	6	4280.9702	-0.0026
5	1	5	4	1	4	4292.1924	-0.0007
5	0	5	4	0	4	4377.8799	-0.0001
7	4	3	7	3	4	4420.5166	0.0012
5	1	5	4	0	4	4465.7130	-0.0011
7	3	5	7	2	6	4518.6590	0.0010
9	2	7	9	1	8	4686.0989	-0.0053

6	4	2	6	3	3	4699.1619	0.0019
5	2	4	4	2	3	4805.5373	-0.0013
3	2	2	2	1	1	4812.2239	0.0000
5	4	1	5	3	2	4875.2343	-0.0030
7	2	6	7	1	7	4953.1889	-0.0010
4	4	0	4	3	1	4964.5347	0.0026
5	4	2	4	4	1	4968.7724	0.0021
5	4	1	4	4	0	4972.4707	-0.0054
5	3	3	4	3	2	4973.3315	-0.0008
7	2	5	6	3	4	4978.9199	0.0000
5	4	2	5	3	3	4990.8682	0.0030
4	4	1	4	3	2	4995.4186	-0.0086
6	4	3	6	3	4	5009.6322	0.0033
6	0	6	5	1	5	5059.7281	-0.0003
5	3	2	4	3	1	5061.7696	-0.0014
6	1	5	5	2	4	5069.5243	0.0016
7	4	4	7	3	5	5075.2467	0.0008
6	1	6	5	1	5	5101.0131	-0.0005
6	0	6	5	0	5	5147.5622	-0.0004
5	1	4	4	1	3	5151.0426	0.0000
6	1	6	5	0	5	5188.8489	0.0012
8	4	5	8	3	6	5212.7414	-0.0027
5	2	3	4	2	2	5311.1149	0.0002
8	1	7	8	0	8	5478.7723	0.0054
4	2	3	3	1	2	5502.2664	-0.0022
10	5	5	10	4	6	5537.3553	0.0004
3	2	1	2	1	2	5556.5152	-0.0010
8	2	7	8	1	8	5675.5714	0.0032
6	2	5	5	2	4	5702.9877	0.0006
10	4	7	10	3	8	5780.4479	0.0087
7	0	7	6	1	6	5881.6532	0.0004
9	5	4	9	4	5	5895.2979	-0.0002
7	1	7	6	1	6	5900.0997	-0.0088
7	0	7	6	0	6	5922.9350	-0.0029
7	1	7	6	0	6	5941.3950	0.0014
6	5	1	5	5	0	5958.5709	-0.0069
6	3	4	5	3	3	5966.1742	-0.0002
6	4	3	5	4	2	5984.9318	-0.0062
6	4	2	5	4	1	6001.1100	0.0028
6	1	5	5	1	4	6026.3638	-0.0028
5	2	4	4	1	3	6107.8851	-0.0013
8	5	3	8	4	4	6141.5002	-0.0031
6	3	3	5	3	2	6177.1857	0.0012
7	1	6	6	2	5	6195.2238	-0.0002
10	3	8	10	2	9	6214.4014	-0.0054
7	5	2	7	4	3	6289.1386	0.0037
9	5	5	9	4	6	6323.3039	-0.0056
8	5	4	8	4	5	6329.6929	0.0055
9	1	8	9	0	9	6329.9005	0.0008

7	5	3	7	4	4	6357.7735	0.0027
6	5	1	6	4	2	6370.2794	-0.0032
6	5	2	6	4	3	6390.1523	-0.0009
6	2	4	5	2	3	6403.8424	0.0032
5	5	0	5	4	1	6412.8083	-0.0038
5	5	1	5	4	2	6416.9459	0.0007
9	2	8	9	1	9	6430.1888	0.0016
8	2	6	7	3	5	6440.3256	-0.0017
3	3	1	2	2	0	6523.4885	0.0016
3	3	0	2	2	1	6564.9201	-0.0016
7	2	6	6	2	5	6572.3242	-0.0015
6	2	5	5	1	4	6659.8341	0.0031
8	0	8	7	1	7	6685.5435	0.0008
8	1	8	7	1	7	6693.5050	-0.0008
8	0	8	7	0	7	6703.9999	0.0015
8	1	8	7	0	7	6711.9669	0.0054
7	1	6	6	1	5	6828.6879	-0.0004
7	3	5	6	3	4	6941.0759	0.0018
7	5	3	6	5	2	6974.3096	0.0008
7	4	4	6	4	3	7006.6938	0.0027
7	4	3	6	4	2	7057.7824	0.0017
7	2	6	6	1	5	7205.7885	-0.0016
8	1	7	7	2	6	7211.1240	0.0043
7	3	4	6	3	3	7336.4297	0.0044
8	2	7	7	2	6	7415.8780	-0.0060
4	3	2	3	2	1	7423.8383	-0.0008
7	2	5	6	2	4	7440.2633	0.0007
9	0	9	8	1	8	7480.5633	0.0036
9	1	9	8	1	8	7483.9107	0.0043
9	0	9	8	0	8	7488.5216	-0.0012
9	1	9	8	0	8	7491.8675	-0.0020
8	1	7	7	1	6	7588.2225	0.0011
4	3	1	3	2	2	7631.0950	0.0003
9	6	3	9	5	4	7693.1714	-0.0017
8	2	7	7	1	6	7792.9781	-0.0077
7	6	1	7	5	2	7817.5414	0.0021
8	3	6	7	3	5	7890.4096	-0.0014

Table S18. Measured frequencies and residuals (in MHz) for the rotational transitions of the $\mathbf{H_2{}^{18}O}$ isotopologue of the isomer **1w-1** of 1,4-NQ-H_2O.

J'	K'_{-1}	K'_{+1}	J''	K''_{-1}	K''_{+1}	ν_{obs}	ν_{obs}- ν_{calc}
3	1	3	2	0	2	3040.7390	0.0069
4	0	4	3	1	3	3139.8559	0.0020
4	0	4	3	0	3	3486.0365	0.0161
4	1	4	3	0	3	3692.6596	-0.0005
4	2	3	3	2	2	3724.2954	-0.0118
4	2	2	3	2	1	3986.2612	-0.0003
2	2	1	1	1	0	4009.3653	-0.0012
4	1	3	3	1	2	4025.4480	0.0005
5	0	5	4	1	4	4033.8746	-0.0007
5	1	5	4	1	4	4145.0170	0.0042
2	2	0	1	1	1	4214.7211	0.0076
5	0	5	4	0	4	4240.5166	0.0017
5	1	5	4	0	4	4351.6491	-0.0033
5	2	4	4	2	3	4617.5299	-0.0113
6	1	5	5	2	4	4730.5660	0.0108
3	2	2	2	1	1	4771.6891	0.0019
5	3	2	4	3	1	4826.7046	-0.0060
6	0	6	5	1	5	4874.3480	-0.0022
6	1	6	5	1	5	4929.8489	-0.0016
5	1	4	4	1	3	4951.7639	0.0028
6	0	6	5	0	5	4985.4812	-0.0064
6	1	6	5	0	5	5040.9905	0.0026
5	2	3	4	2	2	5059.9057	0.0047
3	2	1	2	1	2	5443.1894	0.0036
4	2	3	3	1	2	5443.7634	-0.0027
6	2	5	5	2	4	5487.5956	-0.0069
7	0	7	6	1	6	5678.6200	0.0018
7	1	7	6	1	6	5704.9487	-0.0071
6	3	4	5	3	3	5713.2716	0.0123
7	0	7	6	0	6	5734.1176	-0.0009
7	1	7	6	0	6	5760.4599	0.0038
6	1	5	5	1	4	5814.6505	-0.0033
7	1	6	6	2	5	5853.0000	0.0067
8	2	6	7	3	5	5855.5463	-0.0039
6	3	3	5	3	2	5876.6688	-0.0067
5	2	4	4	1	3	6035.8633	0.0036
6	2	4	5	2	3	6113.6377	0.0008
7	2	6	6	2	5	6333.1186	-0.0022
8	0	8	7	1	7	6461.9074	0.0038
8	1	8	7	1	7	6473.9421	-0.0172
8	0	8	7	0	7	6488.2363	-0.0050
8	1	8	7	0	7	6500.2949	-0.0020
3	3	1	2	2	0	6502.6382	-0.0048
3	3	0	2	2	1	6536.6479	-0.0038
6	2	5	5	1	4	6571.6997	-0.0014
7	1	6	6	1	5	6610.0408	0.0002

7	3	5	6	3	4	6653.4867	-0.0005
7	2	6	6	1	5	7090.1780	0.0099
9	0	9	8	1	8	7234.1564	0.0124
4	3	1	3	2	2	7546.0522	-0.0011

Table S19. Measured frequencies and residuals (in MHz) for the rotational transitions of the isomer **2w-1** of 1,4-NQ-$(H_2O)_2$.

J'	K'_{-1}	K'_{+1}	J''	K''_{-1}	K''_{+1}	ν_{obs}	ν_{obs}- ν_{calc}
3	1	3	2	0	2	2418.6120	-0.0092
4	0	4	3	0	3	2592.8225	-0.0030
8	3	5	8	2	6	2755.1137	0.0100
5	0	5	4	1	4	2861.8056	0.0035
6	1	5	5	2	4	2880.5015	0.0016
4	1	3	3	1	2	2890.8356	-0.0053
4	1	4	3	0	3	2907.3169	0.0048
8	2	6	7	3	5	3050.8027	-0.0149
5	1	5	4	1	4	3070.3900	-0.0060
5	1	5	4	1	4	3070.3900	-0.0060
5	0	5	4	0	4	3176.2852	-0.0035
5	3	2	5	2	3	3268.6932	-0.0146
6	2	5	6	1	6	3289.7789	0.0038
5	2	4	4	2	3	3354.2382	0.0116
5	1	5	4	0	4	3384.8821	-0.0005
4	3	1	4	2	2	3395.7433	0.0087
2	2	1	1	1	0	3412.6793	0.0074
5	3	3	4	3	2	3413.9589	0.0059
5	3	2	4	3	1	3431.4190	0.0157
2	2	0	1	1	1	3531.1214	0.0045
6	0	6	5	1	5	3533.3948	-0.0048
5	2	3	4	2	2	3558.4321	0.0020
4	3	2	4	2	3	3560.7089	0.0037
5	1	4	4	1	3	3585.1365	0.0131
5	3	3	5	2	4	3620.4236	-0.0080
7	2	6	7	1	7	3683.5181	-0.0019
6	0	6	5	0	5	3741.9995	0.0060
7	1	6	6	2	5	3774.5972	-0.0033
7	3	5	7	2	6	3864.7977	-0.0061
6	1	6	5	0	5	3871.2736	0.0007
3	2	2	2	1	1	3982.4103	0.0006
6	2	5	5	2	4	4003.4996	0.0020
9	2	7	8	3	6	4091.5529	0.0037
6	4	2	5	4	1	4099.0946	0.0138
6	3	4	5	3	3	4101.9303	-0.0046
8	2	7	8	1	8	4123.8974	0.0005
6	3	3	5	3	2	4146.8917	-0.0135
10	4	6	10	3	7	4170.2219	0.0031
7	0	7	6	1	6	4172.0287	0.0026
7	1	7	6	1	6	4248.0834	0.0052
6	1	5	5	1	4	4256.2887	-0.0099
7	0	7	6	0	6	4301.3100	0.0045
6	2	4	5	2	3	4316.7894	-0.0020
3	2	1	2	1	2	4360.7727	-0.0076
7	1	7	6	0	6	4377.3587	0.0011
9	4	5	9	3	6	4412.6936	0.0005

4	2	3	3	1	2	4497.5350	-0.0014
8	1	7	7	2	6	4638.0704	0.0027
7	2	6	6	2	5	4641.8087	-0.0144
8	0	8	7	1	7	4785.0223	0.0001
8	1	8	7	1	7	4828.0636	-0.0017
6	4	2	6	3	3	4834.9528	-0.0013
8	0	8	7	0	7	4861.0818	0.0075
8	1	8	7	0	7	4904.1254	0.0080
5	4	2	5	3	3	4905.7134	0.0042
4	4	0	4	3	1	4906.2798	0.0075
4	4	1	4	3	2	4912.1516	-0.0008
8	4	5	8	3	6	4923.1049	-0.0032
5	2	4	4	1	3	4960.9218	-0.0003
9	4	6	9	3	7	4968.0902	-0.0079
7	2	5	6	2	4	5067.9177	-0.0023
10	2	9	10	1	10	5112.6360	0.0059
4	2	2	3	1	3	5306.2014	0.0009
6	2	5	5	1	4	5379.2922	-0.0041
9	0	9	8	1	8	5380.4798	0.0004
9	1	9	8	0	8	5447.1945	0.0026
9	1	8	8	2	7	5452.0485	0.0080
8	1	7	7	1	6	5505.2830	-0.0072
3	3	1	2	2	0	5546.2025	0.0020
3	3	0	2	2	1	5559.0609	-0.0018
7	2	6	6	1	5	5764.8157	-0.0050
10	0	10	9	1	9	5964.8999	0.0133
10	1	10	9	0	9	6001.2906	0.0022
9	1	8	8	1	7	6082.4105	-0.0047
10	5	5	10	4	6	6102.8646	-0.0036
8	2	7	7	1	6	6135.6658	0.0010
9	5	4	9	4	5	6192.5098	-0.0036
4	3	2	3	2	1	6196.9820	0.0016
10	1	9	9	2	8	6207.9045	-0.0040
8	5	3	8	4	4	6248.7866	0.0231
4	3	1	3	2	2	6261.9751	0.0019
7	5	2	7	4	3	6283.0734	-0.0175
7	5	3	7	4	4	6290.9878	-0.0001
6	5	1	6	4	2	6303.5758	0.0010
6	5	2	6	4	3	6305.7786	0.0020
5	2	3	4	1	4	6394.4736	0.0166
9	2	8	8	1	7	6513.6898	-0.0030
11	0	11	10	1	10	6542.7181	0.0022
11	1	11	10	0	10	6562.1790	-0.0006
5	3	3	4	2	2	6803.7393	-0.0180
11	1	10	10	2	9	6908.6983	-0.0087
10	2	9	9	1	8	6918.4633	0.0029
5	3	2	4	2	3	6998.0459	0.0070
6	3	4	5	2	3	7347.2595	-0.0026
12	1	11	11	2	10	7564.8631	-0.0009

6	3	3	5	2	4	7790.7109	-0.0066
7	3	5	6	2	4	7818.2239	-0.0047

Table S20. Measured frequencies and residuals (in MHz) for the rotational transitions of the **1618** isotopologue of the isomer **2w-1** of 1,4-NQ-$(H_2O)_2$.

J'	K'_{-1}	K'_{+1}	J''	K''_{-1}	K''_{+1}	ν_{obs}	ν_{obs}- ν_{calc}
5	0	5	4	1	4	2769.1810	0.0035
5	1	5	4	0	4	3323.8212	-0.0055
2	2	1	1	1	0	3388.6062	-0.0049
6	0	6	5	1	5	3429.7165	0.0131
2	2	0	1	1	1	3500.6823	-0.0004
7	1	6	6	2	5	3596.0364	0.0036
6	1	6	5	0	5	3795.8144	-0.0122
3	2	2	2	1	1	3944.6903	-0.0018
7	0	7	6	1	6	4058.2415	-0.0020
7	1	7	6	0	6	4285.8841	0.0046
3	2	1	2	1	2	4301.7415	-0.0030
8	1	7	7	2	6	4445.8039	0.0009
4	2	3	3	1	2	4448.8667	-0.0016
8	0	8	7	1	7	4660.9887	0.0019
8	1	8	7	0	7	4796.1003	0.0066
5	2	4	4	1	3	4903.6263	-0.0017
4	2	2	3	1	3	5210.3879	0.0007
9	0	9	8	1	8	5245.5059	-0.0007
9	1	8	8	2	7	5252.1701	-0.0064
6	2	5	5	1	4	5314.7963	0.0002
9	1	9	8	0	8	5322.9359	-0.0021
3	3	1	2	2	0	5508.5574	-0.0030
3	3	0	2	2	1	5520.1116	0.0075
7	2	6	6	1	5	5692.9367	0.0025
10	0	10	9	1	9	5818.2129	0.0010
10	1	10	9	0	9	5861.4315	0.0014
10	1	9	9	2	8	6004.5189	-0.0065
8	2	7	7	1	6	6054.0493	-0.0077
4	3	2	3	2	1	6143.0514	0.0136
4	3	1	3	2	2	6201.3803	-0.0019
5	2	3	4	1	4	6252.4396	0.0036
11	0	11	10	1	10	6383.6956	0.0019
11	1	11	10	0	10	6407.3298	0.0024
9	2	8	8	1	7	6418.1250	0.0014
11	1	10	10	2	9	6702.9833	-0.0041
5	3	3	4	2	2	6737.5240	-0.0036
5	3	2	4	2	3	6912.2098	-0.0029
12	0	12	11	1	11	6944.9441	0.0015
12	1	12	11	0	11	6957.6603	0.0021
6	3	4	5	2	3	7273.5735	-0.0074
6	2	4	5	1	5	7443.8255	-0.0035
6	3	3	5	2	4	7673.4489	0.0088

Table S21. Measured frequencies and residuals (in MHz) for the rotational transitions of the **1816** isotopologue of the isomer **2w-1** of 1,4-NQ-(H_2O)$_2$.

J'	K'_{-1}	K'_{+1}	J''	K''_{-1}	K''_{+1}	ν_{obs}	ν_{obs}- ν_{calc}
5	0	5	4	1	4	2781.7368	0.0002
5	1	5	4	0	4	3322.4316	0.0174
2	2	1	1	1	0	3374.7406	0.0008
6	0	6	5	1	5	3441.6776	0.0028
2	2	0	1	1	1	3488.1526	0.0007
7	1	6	6	2	5	3631.3738	0.0007
6	1	6	5	0	5	3796.0537	0.0011
3	2	2	2	1	1	3931.7138	-0.0002
7	0	7	6	1	6	4069.4846	-0.0013
7	1	7	6	0	6	4288.1725	-0.0048
3	2	1	2	1	2	4293.3472	-0.0081
4	2	3	3	1	2	4436.2385	-0.0005
8	1	7	7	2	6	4480.3251	0.0018
8	0	8	7	1	7	4671.7020	0.0012
8	1	8	7	0	7	4800.5218	-0.0001
5	2	4	4	1	3	4890.9053	-0.0012
4	2	2	3	1	3	5208.0812	0.0017
9	0	9	8	1	8	5256.0186	-0.0051
9	1	8	8	2	7	5284.1539	-0.0020
6	2	5	5	1	4	5301.7860	-0.0017
9	1	9	8	0	8	5329.2999	-0.0007
3	3	1	2	2	0	5485.5216	-0.0066
3	3	0	2	2	1	5497.4218	0.0053
7	2	6	6	1	5	5679.8795	-0.0003
10	0	10	9	1	9	5828.8582	0.0021
10	1	10	9	0	9	5869.4455	-0.0070
10	1	9	9	2	8	6032.9101	-0.0025
8	2	7	7	1	6	6041.7821	0.0005
4	3	2	3	2	1	6121.2745	0.0056
4	3	1	3	2	2	6181.3502	-0.0003
5	2	3	4	1	4	6258.4074	-0.0004
11	0	11	10	1	10	6394.7279	0.0032
11	1	11	10	0	10	6416.7539	-0.0076
5	3	3	4	2	2	6715.9909	-0.0013
11	1	10	10	2	9	6727.6561	-0.0025
5	3	2	4	2	3	6895.7873	-0.0005
12	0	12	11	1	11	6956.5511	0.0043
12	1	12	11	0	11	6968.3237	0.0071
6	3	4	5	2	3	7251.0868	-0.0006
6	2	4	5	1	5	7460.0014	0.0035
6	3	3	5	2	4	7662.2664	-0.0008

Table S22. Measured frequencies and residuals (in MHz) for the rotational transitions of the **1818** isotopologue of the isomer **2w-1** of 1,4-NQ-$(H_2O)_2$.

J'	K'_{-1}	K'_{+1}	J''	K''_{-1}	K''_{+1}	ν_{obs}	ν_{obs}- ν_{calc}
5	1	5	4	0	4	3263.9349	-0.0020
6	0	6	5	1	5	3344.6852	0.0029
7	0	7	6	1	6	3962.6469	0.0046
8	0	8	7	1	7	4554.9915	0.0008
9	0	9	8	1	8	5128.9136	-0.0002
10	0	10	9	1	9	5690.6951	-0.0005
11	0	11	10	1	10	6244.9425	0.0015
12	0	12	11	1	11	6794.7043	-0.0013
13	0	13	12	1	12	7341.8892	-0.0027
14	0	14	13	1	13	7887.6329	-0.0005
11	1	11	10	0	10	6271.0597	0.0012
12	1	12	11	0	11	6808.9335	-0.0009
13	1	13	12	0	12	7349.5445	0.0029
14	1	14	13	0	13	7891.7029	0.0020
3	2	1	2	1	2	4235.6345	-0.0029
3	2	2	2	1	1	3892.8974	0.0037
4	2	2	3	1	3	5117.1161	0.0030
4	2	3	3	1	2	4387.0017	0.0106
5	2	3	4	1	4	6125.8224	0.0040
5	2	4	4	1	3	4833.3440	0.0009
6	2	5	5	1	4	5237.2693	-0.0030
7	2	6	6	1	5	5608.4365	-0.0055
8	2	7	7	1	6	5961.6077	-0.0042
9	2	8	8	1	7	6315.5113	0.0067
9	1	8	8	2	7	5098.2140	-0.0053
10	1	9	9	2	8	5842.3512	-0.0031
3	3	1	2	2	0	5444.6373	-0.0026
3	3	0	2	2	1	5455.4090	0.0019
4	3	2	3	2	1	6065.0361	-0.0035
5	3	3	4	2	2	6647.8925	-0.0041
5	3	2	4	2	3	6810.9807	0.0002
6	3	3	5	2	4	7549.2843	0.0016
6	3	4	5	2	3	7175.4058	-0.0024

Table S23. Measured frequencies and residuals (in MHz) for the rotational transitions of the isomer **2w-2** of 1,4-NQ-$(H_2O)_2$.

J'	K'_{-1}	K'_{+1}	J''	K''_{-1}	K''_{+1}	ν_{obs}	$\nu_{obs}-\nu_{calc}$
6	3	3	6	2	4	2321.4296	-0.0053
5	3	2	5	2	3	2509.4579	-0.0042
4	0	4	3	1	3	2535.3164	0.0021
4	1	4	3	1	3	2694.4929	0.0027
4	3	1	4	2	2	2698.3685	-0.0123
4	0	4	3	0	3	2805.0302	0.0128
10	4	6	10	3	7	2955.6946	-0.0007
4	1	4	3	0	3	2964.1931	-0.0002
4	3	2	4	2	3	3016.2915	0.0034
5	3	3	5	2	4	3133.6884	-0.0012
9	4	5	9	3	6	3155.4272	0.0148
2	2	1	1	1	0	3202.8539	0.0031
4	1	3	3	1	2	3244.4861	0.0021
5	0	5	4	1	4	3252.2415	0.0014
6	3	4	6	2	5	3319.5568	-0.0007
5	1	5	4	1	4	3336.8148	0.0053
6	2	5	6	1	6	3345.3257	0.0094
2	2	0	1	1	1	3369.9642	0.0064
5	0	5	4	0	4	3411.4157	-0.0002
8	4	4	8	3	5	3423.7476	0.0001
5	1	5	4	0	4	3495.9831	-0.0023
7	3	5	7	2	6	3582.7460	0.0003
5	2	4	4	2	3	3720.2645	-0.0039
3	2	2	2	1	1	3816.3998	-0.0023
6	1	5	5	2	4	3837.1338	0.0054
5	3	3	4	3	2	3837.6698	-0.0001
6	4	2	6	3	3	3877.9535	0.0004
8	3	6	8	2	7	3925.4589	-0.0080
6	0	6	5	1	5	3926.3467	0.0002
6	1	6	5	1	5	3968.0628	-0.0016
5	1	4	4	1	3	3988.9014	0.0020
5	4	1	5	3	2	3996.9226	-0.0114
6	0	6	5	0	5	4010.9210	0.0050
5	4	2	5	3	3	4070.9760	0.0017
6	4	3	6	3	4	4080.0944	0.0019
5	2	3	4	2	2	4083.1877	0.0017
8	4	5	8	3	6	4206.6158	-0.0116
4	2	3	3	1	2	4356.7403	-0.0009
9	4	6	9	3	7	4358.4283	-0.0021
3	2	1	2	1	2	4363.5093	0.0059
6	2	5	5	2	4	4420.1104	-0.0002
7	0	7	6	1	6	4571.9838	-0.0011
7	1	7	6	1	6	4591.5438	-0.0001
6	3	4	5	3	3	4605.9718	-0.0067
7	0	7	6	0	6	4613.7034	0.0006
7	1	7	6	0	6	4633.2614	-0.0004

6	1	5	5	1	4	4680.7527	-0.0019
7	1	6	6	2	5	4734.7013	0.0005
6	3	3	5	3	2	4743.5126	0.0015
5	2	4	4	1	3	4832.5248	-0.0008
6	2	4	5	2	3	4931.5399	0.0016
8	5	3	8	4	4	5057.7699	0.0107
7	2	6	6	2	5	5099.7997	-0.0015
7	5	2	7	4	3	5153.6031	0.0017
3	3	1	2	2	0	5193.7247	0.0039
7	5	3	7	4	4	5194.0478	0.0009
8	0	8	7	1	7	5201.3649	-0.0028
6	5	1	6	4	2	5206.6924	0.0002
8	1	8	7	1	7	5210.2166	0.0026
6	5	2	6	4	3	5218.2899	-0.0068
8	0	8	7	0	7	5220.9285	0.0018
3	3	0	2	2	1	5221.9816	0.0035
8	1	8	7	0	7	5229.7721	-0.0009
6	2	5	5	1	4	5263.7353	-0.0015
7	1	6	6	1	5	5317.6841	0.0010
7	3	5	6	3	4	5362.9900	0.0005
4	2	2	3	1	3	5542.0936	0.0181
8	1	7	7	2	6	5551.2960	-0.0038
7	3	4	6	3	3	5627.7974	-0.0060
7	2	6	6	1	5	5682.7807	-0.0028
7	2	5	6	2	4	5743.0645	-0.0018
9	0	9	8	1	8	5822.3112	0.0009
9	1	9	8	1	8	5826.2038	-0.0025
9	0	9	8	0	8	5831.1553	-0.0013
9	1	9	8	0	8	5835.0524	-0.0002
4	3	2	3	2	1	5895.7150	0.0037
8	1	7	7	1	6	5916.3941	-0.0061
4	3	1	3	2	2	6037.4573	0.0041
8	3	6	7	3	5	6103.0897	-0.0068
8	2	7	7	1	6	6125.4765	0.0008
9	1	8	8	2	7	6293.2363	-0.0029
10	0	10	9	1	9	6439.1471	-0.0016
10	0	10	9	0	9	6443.0402	-0.0045
10	1	10	9	0	9	6444.7249	-0.0007
9	1	8	8	1	7	6502.3169	0.0022
5	3	3	4	2	2	6516.1842	0.0002
8	3	5	7	3	4	6526.0071	-0.0077
9	2	8	8	1	7	6613.8029	0.0020
9	3	7	8	3	6	6822.3501	0.0030
5	3	2	4	2	3	6930.4199	-0.0022
10	1	9	9	2	8	6980.4153	-0.0026
6	3	4	5	2	3	7038.9729	-0.0036
11	0	11	10	1	10	7054.0872	-0.0001
11	1	11	10	1	10	7054.7950	-0.0057
11	0	11	10	0	10	7055.7741	0.0059

11	1	11	10	0	10	7056.4823	0.0007
10	1	9	9	1	8	7091.9047	0.0006
10	2	9	9	1	8	7148.2734	0.0017
7	3	5	6	2	4	7470.4255	-0.0021
11	1	10	10	2	9	7633.1110	-0.0036
12	0	12	11	1	11	7668.1851	0.0038
12	1	12	11	1	11	7668.4821	0.0020
12	1	12	11	0	11	7669.1993	0.0057
11	1	10	10	1	9	7689.4870	0.0048
8	3	6	7	2	5	7830.4521	-0.0057
6	3	3	5	2	4	7953.6647	-0.0002

Table S24. Measured frequencies and residuals (in MHz) for the rotational transitions of the **1618** isotopologue of the isomer **2w-2** of 1,4-NQ-$(H_2O)_2$.

J'	K'_{-1}	K'_{+1}	J''	K''_{-1}	K''_{+1}	ν_{obs}	$\nu_{obs}-\nu_{calc}$
4	1	4	3	0	3	2915.1436	-0.0036
5	0	5	4	1	4	3178.3996	0.0026
5	1	5	4	0	4	3434.5193	-0.0005
6	0	6	5	1	5	3842.3683	-0.0017
6	1	6	5	0	5	3977.5101	-0.0021
4	2	3	3	1	2	4300.5303	-0.0045
7	0	7	6	1	6	4477.5415	0.0034
7	1	7	6	0	6	4544.3236	0.0017
7	1	6	6	2	5	4602.9737	0.0039
5	2	4	4	1	3	4768.4047	-0.0024
8	0	8	7	1	7	5095.8786	-0.0054
8	1	8	7	0	7	5127.4100	0.0020
3	3	1	2	2	0	5138.9253	-0.0065
6	2	5	5	1	4	5191.7127	-0.0019
8	1	7	7	2	6	5413.2685	-0.0033
7	2	6	6	1	5	5600.4534	-0.0058
9	0	9	8	1	8	5705.3140	0.0031
9	1	9	8	0	8	5719.7127	0.0041
4	3	2	3	2	1	5827.2332	0.0062
4	3	1	3	2	2	5959.2716	0.0084
8	2	7	7	1	6	6029.0462	0.0003
9	1	8	8	2	7	6149.7962	-0.0055
10	0	10	9	1	9	6310.3122	0.0004
10	1	10	9	0	9	6316.7275	0.0005
5	3	3	4	2	2	6438.3619	0.0067
9	2	8	8	1	7	6500.5378	0.0092
5	3	2	4	2	3	6825.1079	0.0024
10	1	9	9	2	8	6830.4233	-0.0026
11	0	11	10	1	10	6913.2246	0.0024
6	3	4	5	2	3	6955.5576	0.0009
10	2	9	9	1	8	7017.7434	0.0053
7	3	5	6	2	4	7383.5171	-0.0087
12	0	12	11	1	11	7515.1852	0.0014
12	1	12	11	0	11	7516.3839	-0.0065
6	3	3	5	2	4	7812.1043	-0.0051

Table S25. Measured frequencies and residuals (in MHz) for the rotational transitions of the **1816** isotopologue of the isomer **2w-2** of 1,4-NQ-$(H_2O)_2$.

J'	K'_{-1}	K'_{+1}	J"	K''_{-1}	K''_{+1}	ν_{obs}	ν_{obs}- ν_{calc}
4	1	4	3	0	3	2913.4068	0.0001
5	0	5	4	1	4	3160.4833	-0.0007
6	0	6	5	1	5	3824.8511	0.0020
6	1	6	5	0	5	3968.9029	0.0116
4	2	3	3	1	2	4310.3425	-0.0085
7	0	7	6	1	6	4459.8314	-0.0022
7	1	7	6	0	6	4532.0184	-0.0018
7	1	6	6	2	5	4557.4570	0.0052
5	2	4	4	1	3	4777.8975	-0.0043
8	0	8	7	1	7	5077.3557	-0.0014
8	1	8	7	0	7	5111.9080	0.0001
3	3	1	2	2	0	5160.2566	0.0034
3	3	0	2	2	1	5185.4269	-0.0003
6	2	5	5	1	4	5200.4375	-0.0010
8	1	7	7	2	6	5372.7059	0.0050
7	2	6	6	1	5	5606.5175	-0.0080
9	0	9	8	1	8	5685.4679	-0.0043
9	1	9	8	0	8	5701.4714	0.0002
4	3	2	3	2	1	5846.4305	-0.0054
4	3	1	3	2	2	5972.9100	-0.0108
8	2	7	7	1	6	6029.8095	0.0031
9	1	8	8	2	7	6114.1975	-0.0093
10	0	10	9	1	9	6288.8440	-0.0011
10	1	10	9	0	9	6296.0774	0.0054
5	3	3	4	2	2	6457.8047	0.0016
9	2	8	8	1	7	6494.2475	0.0019
10	1	9	9	2	8	6798.3705	-0.0075
5	3	2	4	2	3	6828.9358	0.0150
11	0	11	10	1	10	6889.9485	0.0058
11	1	11	10	0	10	6893.1477	0.0029
6	3	4	5	2	3	6977.0118	0.0025
10	2	9	9	1	8	7004.4507	0.0010
11	1	10	10	2	9	7444.6053	-0.0048
12	0	12	11	1	11	7489.9973	0.0060
12	1	12	11	0	11	7491.3898	0.0016
11	2	10	10	1	9	7550.9833	-0.0117
8	3	6	7	2	5	7766.4606	0.0067

Table S26. Measured frequencies and residuals (in MHz) for the rotational transitions of the isomer **3w-1** of 1,4-NQ-$(H_2O)_3$.

J'	K'_{-1}	K'_{+1}	J''	K''_{-1}	K''_{+1}	ν_{obs}	ν_{obs}- ν_{calc}
4	0	4	3	0	3	2094.2308	0.0005
4	2	2	3	2	1	2261.0455	0.0022
5	0	5	4	1	4	2318.0986	0.0015
4	1	3	3	1	2	2327.1245	0.0017
6	1	5	5	2	4	2341.1289	0.0038
4	1	4	3	0	3	2343.3906	-0.0023
5	1	5	4	1	4	2483.8026	-0.0010
5	0	5	4	0	4	2567.2600	0.0002
5	2	4	4	2	3	2705.8937	-0.0001
2	2	1	1	1	0	2714.2144	-0.0001
5	1	5	4	0	4	2732.9748	0.0086
2	2	0	1	1	1	2806.8537	-0.0002
6	0	6	5	1	5	2860.5777	0.0027
5	2	3	4	2	2	2864.9464	0.0018
5	1	4	4	1	3	2886.8291	0.0026
6	1	6	5	1	5	2963.5646	-0.0031
6	0	6	5	0	5	3026.2824	0.0010
7	1	6	6	2	5	3057.9881	0.0067
6	1	6	5	0	5	3129.2739	-0.0002
6	2	5	5	2	4	3230.2866	-0.0021
6	4	3	5	4	2	3303.2593	-0.0064
6	3	4	5	3	3	3306.9370	0.0018
6	3	3	5	3	2	3341.6954	0.0015
7	0	7	6	1	6	3377.1762	-0.0012
6	1	5	5	1	4	3428.5946	0.0010
7	1	7	6	1	6	3437.9409	-0.0006
3	2	1	2	1	2	3472.0770	0.0018
6	2	4	5	2	3	3474.5777	0.0030
7	0	7	6	0	6	3480.1674	-0.0027
7	1	7	6	0	6	3540.9390	0.0049
4	2	3	3	1	2	3595.5160	-0.0080
8	1	7	7	2	6	3751.3186	0.0031
7	3	5	6	3	4	3859.8456	0.0019
7	4	3	6	4	2	3866.5364	-0.0029
8	0	8	7	1	7	3873.5760	0.0008
8	1	8	7	1	7	3908.0641	-0.0011
7	3	4	6	3	3	3933.6351	0.0044
8	0	8	7	0	7	3934.3353	-0.0039
7	1	6	6	1	5	3947.1459	0.0009
8	1	8	7	0	7	3968.8271	-0.0021
5	2	4	4	1	3	3974.2934	-0.0016
7	2	5	6	2	4	4078.7555	0.0004
4	2	2	3	1	3	4227.6387	0.0013
8	2	7	7	2	6	4252.9099	0.0007
6	2	5	5	1	4	4317.7548	-0.0024
9	0	9	8	1	8	4356.0935	0.0007

9	1	9	8	1	8	4375.1102	-0.0032
9	0	9	8	0	8	4390.5803	-0.0025
9	1	8	8	2	7	4406.2688	0.0042
3	3	1	2	2	0	4407.5320	0.0038
8	5	4	7	5	3	4408.3927	-0.0002
8	3	6	7	3	5	4408.8364	0.0022
9	1	9	8	0	8	4409.6072	0.0038
3	3	0	2	2	1	4417.5261	0.0019
8	4	5	7	4	4	4423.3819	0.0003
8	4	4	7	4	3	4435.3958	-0.0011
8	1	7	7	1	6	4439.4949	-0.0004
8	3	5	7	3	4	4543.3619	0.0006
7	2	6	6	1	5	4635.3250	0.0002
8	2	6	7	2	5	4669.1042	0.0006
9	2	8	8	2	7	4750.4916	0.0023
10	0	10	9	1	9	4829.8329	-0.0013
10	1	10	9	1	9	4840.0933	-0.0019
10	0	10	9	0	9	4848.8521	-0.0027
10	1	10	9	0	9	4859.1108	-0.0050
9	1	8	8	1	7	4907.8587	0.0005
4	3	2	3	2	1	4933.1227	0.0058
8	2	7	7	1	6	4941.0876	-0.0014
9	3	7	8	3	6	4951.6787	0.0022
9	5	5	8	5	4	4969.1556	-0.0035
9	5	4	8	5	3	4970.5602	-0.0029
4	3	1	3	2	2	4983.6213	-0.0067
9	4	6	8	4	5	4986.3288	-0.0017
9	4	5	8	4	4	5014.0707	0.0004
10	1	9	9	2	8	5015.8379	0.0040
5	2	3	4	1	4	5094.7509	0.0061
9	3	6	8	3	5	5167.2147	-0.0008
10	2	9	9	2	8	5239.4585	0.0034
9	2	7	8	2	6	5239.9944	0.0027
9	2	8	8	1	7	5252.0835	0.0005
11	0	11	10	1	10	5298.3324	-0.0007
11	1	11	10	1	10	5303.7700	-0.0035
11	0	11	10	0	10	5308.5930	-0.0011
11	1	11	10	0	10	5314.0300	-0.0045
10	1	9	9	1	8	5360.0598	0.0012
5	3	3	4	2	2	5424.4538	-0.0015
10	5	6	9	5	5	5532.9701	-0.0055
10	5	5	9	5	4	5536.8193	-0.0030
10	4	7	9	4	6	5549.3992	0.0032
5	3	2	4	2	3	5575.4677	-0.0089
11	1	10	10	2	9	5582.1323	0.0048
10	2	9	9	1	8	5583.6818	0.0020
10	4	6	9	4	5	5606.1399	-0.0029
11	2	10	10	2	9	5720.8779	0.0050
12	0	12	11	1	11	5763.8292	0.0020

12	1	12	11	1	11	5766.6684	-0.0032
12	0	12	11	0	11	5769.2648	-0.0028
12	1	12	11	0	11	5772.1131	0.0011
10	2	8	9	2	7	5786.8805	0.0010
11	1	10	10	1	9	5805.7527	0.0040
6	3	4	5	2	3	5866.4439	-0.0020
11	2	10	10	1	9	5944.4962	0.0021
4	4	1	3	3	0	6067.8492	-0.0013
4	4	0	3	3	1	6068.5504	0.0023
6	2	4	5	1	5	6085.5208	0.0049
11	5	7	10	5	6	6099.6819	-0.0018
11	5	6	10	5	5	6109.0119	-0.0041
11	4	8	10	4	7	6110.5653	-0.0011
12	1	11	11	2	10	6113.1508	0.0033
12	2	11	11	2	10	6196.1341	0.0043
6	3	3	5	2	4	6211.2755	-0.0012
11	4	7	10	4	6	6214.9695	-0.0046
13	0	13	12	1	12	6227.6491	-0.0039
13	1	13	12	1	12	6229.1189	-0.0041
13	0	13	12	0	12	6230.4957	-0.0017
13	1	13	12	0	12	6231.9682	0.0008
7	3	5	6	2	4	6251.7122	-0.0027
12	1	11	11	1	10	6251.8990	0.0062
11	2	9	10	2	8	6305.9014	0.0002
12	2	11	11	1	10	6334.8788	0.0037
8	3	6	7	2	5	6581.7911	-0.0028
5	4	2	4	3	1	6613.1272	0.0040
5	4	1	4	3	2	6618.0532	0.0043
13	1	12	12	2	11	6618.4847	0.0050
13	2	12	12	2	11	6666.6903	-0.0054
12	4	9	11	4	8	6667.5417	-0.0023
12	5	8	11	5	7	6668.7294	-0.0089
12	5	7	11	5	6	6689.2019	-0.0114
14	0	14	13	1	13	6690.5701	-0.0057
14	1	14	13	1	13	6691.3202	-0.0080
14	1	14	13	0	13	6692.7984	0.0002
13	2	12	12	1	11	6749.6822	0.0042
12	2	10	11	2	9	6795.4265	0.0006
12	4	8	11	4	7	6841.5324	-0.0113
9	3	7	8	2	6	6864.3646	-0.0022
7	3	4	6	2	5	6914.6167	-0.0020
14	1	13	13	2	12	7106.5471	0.0060
10	3	8	9	2	7	7110.8335	-0.0003
14	2	13	13	2	12	7133.9174	-0.0008
6	4	3	5	3	2	7150.5240	-0.0016
15	0	15	14	1	14	7153.0141	-0.0059
15	1	15	14	1	14	7153.3931	-0.0088
15	0	15	14	0	14	7153.7678	-0.0046
15	1	15	14	0	14	7154.1499	-0.0044

14	1	13	13	1	12	7154.7632	0.0061
6	4	2	5	3	3	7170.2938	-0.0058
14	2	13	13	1	12	7182.1385	0.0043
7	2	5	6	1	6	7200.7147	0.0114
13	2	11	12	2	10	7258.5032	0.0022
11	3	9	10	2	8	7335.7307	0.0032
12	3	10	11	2	9	7556.6645	0.0089
15	1	14	14	2	13	7583.6486	0.0051
15	2	14	14	2	13	7598.9064	0.0069
15	2	14	14	1	13	7626.2841	0.0075
7	4	4	6	3	3	7670.8815	-0.0072
14	2	12	13	2	11	7703.4284	0.0070
8	3	5	7	2	6	7711.8147	-0.0041
7	4	3	6	3	4	7729.9019	-0.0019
13	3	11	12	2	10	7792.7442	0.0110

Table S27. Measured frequencies and residuals (in MHz) for the rotational transitions of the **161816** isotopologue of the isomer **3w-1** of 1,4-NQ-$(H_2O)_3$.

J'	K'_{-1}	K'_{+1}	J''	K''_{-1}	K''_{+1}	ν_{obs}	$\nu_{obs}- \nu_{calc}$
6	0	6	5	1	5	2786.1068	0.0009
6	1	6	5	0	5	3080.3483	-0.0051
7	0	7	6	1	6	3296.6367	-0.0007
7	1	7	6	0	6	3480.4378	-0.0029
4	2	3	3	1	2	3570.7023	-0.0059
8	0	8	7	1	7	3786.7591	0.0003
8	1	8	7	0	7	3896.3634	-0.0037
5	2	4	4	1	3	3944.0655	-0.0031
9	0	9	8	1	8	4262.3635	-0.0019
6	2	5	5	1	4	4283.1912	-0.0057
9	1	9	8	0	8	4325.4781	-0.0016
3	3	1	2	2	0	4395.2749	-0.0077
3	3	0	2	2	1	4404.1986	-0.0095
7	2	6	6	1	5	4596.1969	-0.0052
10	0	10	9	1	9	4728.5026	-0.0023
10	1	10	9	0	9	4763.8955	-0.0016
8	2	7	7	1	6	4895.4111	-0.0023
4	3	2	3	2	1	4909.5135	-0.0042
4	3	1	3	2	2	4954.6249	-0.0058
9	2	8	8	1	7	5196.2994	0.0007
11	1	11	10	0	10	5208.2676	0.0005
5	3	3	4	2	2	5392.7566	-0.0042
10	2	9	9	1	8	5513.8916	0.0048
5	3	2	4	2	3	5527.8741	0.0026
12	0	12	11	1	11	5645.7095	-0.0002
12	1	12	11	0	11	5656.2195	-0.0005
6	3	4	5	2	3	5830.5636	-0.0028
11	2	10	10	1	9	5858.0640	-0.0005
4	4	1	3	3	0	6052.7199	-0.0036
4	4	0	3	3	1	6053.3006	-0.0097
13	0	13	12	1	12	6100.6384	-0.0002
13	1	13	12	0	12	6106.2451	0.0001
6	3	3	5	2	4	6140.0335	0.0214
7	3	5	6	2	4	6214.9463	0.0032
12	2	11	11	1	10	6231.1982	0.0056
8	3	6	7	2	5	6546.0856	0.0009
14	0	14	13	1	13	6554.4779	-0.0002
14	1	14	13	0	13	6557.4355	-0.0006
5	4	2	4	3	1	6584.7027	-0.0026
5	4	1	4	3	2	6588.8473	-0.0014
13	2	12	12	1	11	6629.6732	0.0003
7	3	4	6	2	5	6812.1050	0.0048
9	3	7	8	2	6	6830.2561	0.0082
15	0	15	14	1	14	7007.7263	0.0023
15	1	15	14	0	14	7009.2698	-0.0004
14	2	13	13	1	12	7047.3813	-0.0019

10	3	8	9	2	7	7077.3478	0.0139
6	4	3	5	3	2	7109.9302	0.0009
6	4	2	5	3	3	7126.5737	0.0002
15	2	14	14	1	13	7478.3374	-0.0074
8	3	5	7	2	6	7568.3079	-0.0013
7	4	4	6	3	3	7620.5091	0.0048
7	4	3	6	3	4	7670.2729	0.0055

Table S28. Measured frequencies and residuals (in MHz) for the rotational transitions of the **161618** isotopologue of the isomer **3w-1** of 1,4-NQ-$(H_2O)_3$.

J'	K'_{-1}	K'_{+1}	J''	K''_{-1}	K''_{+1}	ν_{obs}	$\nu_{obs}-\nu_{calc}$
6	0	6	5	1	5	2814.5549	0.0013
6	1	6	5	0	5	3079.0588	-0.0029
7	0	7	6	1	6	3322.8712	-0.0014
7	1	7	6	0	6	3484.0818	-0.0029
4	2	3	3	1	2	3538.1441	-0.0025
8	0	8	7	1	7	3811.3056	-0.0003
8	1	8	7	0	7	3905.0845	-0.0003
5	2	4	4	1	3	3910.8163	-0.0035
6	2	5	5	1	4	4248.7413	-0.0052
9	0	9	8	1	8	4286.0755	-0.0012
3	3	1	2	2	0	4337.3894	-0.0053
9	1	9	8	0	8	4338.7601	-0.0009
3	3	0	2	2	1	4347.2260	-0.0029
7	2	6	6	1	5	4561.1845	-0.0029
10	0	10	9	1	9	4752.2058	-0.0023
10	1	10	9	0	9	4781.0387	0.0001
4	3	2	3	2	1	4854.5382	-0.0017
8	2	7	7	1	6	4862.0032	-0.0022
4	3	1	3	2	2	4904.2350	0.0012
9	2	8	8	1	7	5167.9600	-0.0002
11	0	11	10	1	10	5213.1782	0.0000
11	1	11	10	0	10	5228.6400	0.0019
5	3	3	4	2	2	5337.9875	-0.0003
5	3	2	4	2	3	5486.5713	0.0040
10	2	9	9	1	8	5494.1847	0.0029
12	0	12	11	1	11	5671.1895	-0.0003
12	1	12	11	0	11	5679.3484	0.0011
6	3	4	5	2	3	5772.8785	-0.0029
11	2	10	10	1	9	5849.1563	0.0034
4	4	1	3	3	0	5971.3106	-0.0035
4	4	0	3	3	1	5971.9950	-0.0053
6	3	3	5	2	4	6112.1497	0.0075
13	0	13	12	1	12	6127.5636	0.0051
13	1	13	12	0	12	6131.8012	-0.0055
7	3	5	6	2	4	6151.9604	-0.0006
12	2	11	11	1	10	6233.2170	-0.0061
8	3	6	7	2	5	6476.7279	-0.0014
5	4	2	4	3	1	6507.8211	-0.0030
5	4	1	4	3	2	6512.6663	-0.0030
14	0	14	13	1	13	6583.0426	0.0048
14	1	14	13	0	13	6585.2301	0.0041
13	2	12	12	1	11	6641.3285	-0.0037
9	3	7	8	2	6	6754.7466	0.0028
7	3	4	6	2	5	6804.1745	0.0047
10	3	8	9	2	7	6997.2335	0.0139
6	4	3	5	3	2	7036.5930	0.0007

15	0	15	14	1	14	7038.0549	0.0095
15	1	15	14	0	14	7039.1668	0.0046
6	4	2	5	3	3	7056.0463	0.0029
14	2	13	13	1	12	7066.8190	-0.0006
15	2	14	14	1	13	7503.7904	-0.0215
7	4	4	6	3	3	7548.6021	0.0030
8	3	5	7	2	6	7588.5461	-0.0002
7	4	3	6	3	4	7606.6552	0.0039

Table S29. Measured frequencies and residuals (in MHz) for the rotational transitions of the **181616** isotopologue of the isomer **3w-1** of 1,4-NQ-$(H_2O)_3$.

J'	K'_{-1}	K'_{+1}	J''	K''_{-1}	K''_{+1}	ν_{obs}	ν_{obs}- ν_{calc}
6	0	6	5	1	5	2818.4357	0.0031
6	1	6	5	0	5	3078.1316	-0.0042
7	0	7	6	1	6	3326.2541	-0.0006
7	1	7	6	0	6	3483.8794	-0.0016
4	2	3	3	1	2	3531.9514	-0.0051
8	0	8	7	1	7	3814.2970	0.0005
5	2	4	4	1	3	3904.4105	-0.0023
8	1	8	7	0	7	3905.6056	-0.0006
6	2	5	5	1	4	4242.0438	-0.0104
9	0	9	8	1	8	4288.8232	-0.0026
3	3	1	2	2	0	4326.8862	-0.0068
3	3	0	2	2	1	4336.8820	-0.0021
9	1	9	8	0	8	4339.9089	-0.0011
7	2	6	6	1	5	4554.3407	-0.0034
10	0	10	9	1	9	4754.8525	-0.0018
10	1	10	9	0	9	4782.6944	0.0001
4	3	2	3	2	1	4844.3788	-0.0011
8	2	7	7	1	6	4855.4072	-0.0007
4	3	1	3	2	2	4894.8654	0.0012
9	2	8	8	1	7	5162.2081	0.0008
11	0	11	10	1	10	5215.8268	-0.0019
11	1	11	10	0	10	5230.6978	0.0012
5	3	3	4	2	2	5327.7096	-0.0024
10	2	9	9	1	8	5489.8520	0.0026
12	0	12	11	1	11	5673.9163	-0.0034
12	1	12	11	0	11	5681.7326	-0.0004
6	3	4	5	2	3	5761.9781	0.0002
11	2	10	10	1	9	5846.5484	0.0040
4	4	1	3	3	0	5956.5734	-0.0067
4	4	0	3	3	1	5957.2779	-0.0063
6	3	3	5	2	4	6106.3559	0.0063
13	0	13	12	1	12	6130.4140	-0.0029
13	1	13	12	0	12	6134.4695	0.0002
12	2	11	11	1	10	6232.3043	0.0037
8	3	6	7	2	5	6463.6840	0.0011
5	4	2	4	3	1	6493.7036	-0.0038
5	4	1	4	3	2	6498.6780	-0.0007
14	0	14	13	1	13	6586.0555	0.0005
14	1	14	13	0	13	6588.1387	0.0047
13	2	12	12	1	11	6641.8363	-0.0017
9	3	7	8	2	6	6740.6339	0.0061
7	3	4	6	2	5	6801.6672	0.0049
10	3	8	9	2	7	6982.3461	0.0109
6	4	3	5	3	2	7022.9199	0.0018
15	0	15	14	1	14	7041.2417	0.0019
15	1	15	14	0	14	7042.2960	-0.0006

6	4	2	5	3	3	7042.8758	0.0028
14	2	13	13	1	12	7068.4326	-0.0011
15	2	14	14	1	13	7506.2418	-0.0072
7	4	4	6	3	3	7534.9965	0.0027
8	3	5	7	2	6	7590.7119	0.0006
7	4	3	6	3	4	7594.5352	0.0048

Table S30. Measured frequencies and residuals (in MHz) for the rotational transitions of the **161818** isotopologue of the isomer **3w-1** of 1,4-NQ-$(H_2O)_3$.

J'	K'_{-1}	K'_{+1}	J''	K''_{-1}	K''_{+1}	ν_{obs}	$\nu_{obs}-\nu_{calc}$
6	0	6	5	1	5	2743.5311	0.0053
6	1	6	5	0	5	3032.2157	0.0008
7	0	7	6	1	6	3246.0206	0.0036
7	1	7	6	0	6	3426.2039	-0.0021
8	0	8	7	1	7	3728.4301	0.0002
8	1	8	7	0	7	3835.7985	0.0015
9	0	9	8	1	8	4196.5813	0.0013
6	2	5	5	1	4	4215.2987	-0.0015
9	1	9	8	0	8	4258.3580	0.0032
3	3	0	2	2	1	4333.8108	0.0076
7	2	6	6	1	5	4523.3844	0.0011
10	0	10	9	1	9	4655.4366	-0.0004
10	1	10	9	0	9	4690.0502	-0.0006
8	2	7	7	1	6	4817.9499	-0.0015
4	3	2	3	2	1	4831.2868	-0.0045
4	3	1	3	2	2	4875.8413	-0.0025
11	0	11	10	1	10	5108.5780	-0.0067
9	2	8	8	1	7	5114.2733	-0.0010
11	1	11	10	0	10	5127.5858	-0.0007
5	3	3	4	2	2	5306.9914	-0.0080
10	2	9	9	1	8	5427.1536	-0.0026
12	0	12	11	1	11	5558.3619	-0.0088
12	1	12	11	0	11	5568.6306	-0.0032
6	3	4	5	2	3	5737.8610	-0.0161
11	2	10	10	1	9	5766.2945	0.0032
4	4	1	3	3	0	5955.8703	0.0001
13	0	13	12	1	12	6006.2306	-0.0093
6	3	3	5	2	4	6043.4422	0.0084
7	3	5	6	2	4	6116.0731	-0.0050
12	2	11	11	1	10	6133.9453	0.0044
14	0	14	13	1	13	6453.0566	0.0127
5	4	2	4	3	1	6479.6930	-0.0011
5	4	1	4	3	2	6483.7881	-0.0059
13	2	12	12	1	11	6526.5175	0.0049
7	3	4	6	2	5	6705.6625	0.0036
9	3	7	8	2	6	6721.3458	0.0021
15	0	15	14	1	14	6899.2639	-0.0041
15	1	15	14	0	14	6900.7749	0.0006
10	3	8	9	2	7	6964.4003	0.0037
6	4	3	5	3	2	6996.8361	0.0005
6	4	2	5	3	3	7013.3015	-0.0034
8	3	5	7	2	6	7450.9506	-0.0041
7	4	4	6	3	3	7499.4993	0.0070
7	4	3	6	3	4	7548.7315	0.0019

Table S31. Measured frequencies and residuals (in MHz) for the rotational transitions of the **181618** isotopologue of the isomer **3w-1** of 1,4-NQ-(H_2O)$_3$.

J'	K'_{-1}	K'_{+1}	J''	K''_{-1}	K''_{+1}	ν_{obs}	ν_{obs}- ν_{calc}
6	0	6	5	1	5	2775.0347	-0.0005
6	1	6	5	0	5	3028.9893	0.0036
7	1	7	6	0	6	3428.5243	-0.0126
4	2	3	3	1	2	3473.8925	0.0055
8	0	8	7	1	7	3754.7942	0.0027
8	1	8	7	0	7	3843.8106	-0.0052
6	2	5	5	1	4	4172.5224	-0.0009
9	0	9	8	1	8	4221.7089	0.0012
3	3	1	2	2	0	4254.8038	-0.0099
7	2	6	6	1	5	4479.7822	-0.0126
10	0	10	9	1	9	4680.3005	-0.0049
10	1	10	9	0	9	4707.3714	0.0020
4	3	2	3	2	1	4764.2129	0.0042
4	3	1	3	2	2	4814.1686	-0.0034
9	2	8	8	1	7	5078.3759	0.0028
11	0	11	10	1	10	5133.9692	0.0034
11	1	11	10	0	10	5148.4020	0.0035
5	3	3	4	2	2	5239.8217	-0.0071
5	3	2	4	2	3	5389.1602	0.0072
10	2	9	9	1	8	5401.3052	0.0039
12	0	12	11	1	11	5584.8211	0.0069
12	1	12	11	0	11	5592.3864	-0.0014
6	3	4	5	2	3	5666.9840	0.0035
6	3	3	5	2	4	6007.7023	0.0040
13	0	13	12	1	12	6034.1108	0.0001
13	1	13	12	0	12	6038.0369	0.0037
7	3	5	6	2	4	6038.7073	0.0008
12	2	11	11	1	10	6133.1217	-0.0126
8	3	6	7	2	5	6356.7843	-0.0036
5	4	2	4	3	1	6386.0762	-0.0066
14	1	14	13	0	13	6484.5846	0.0029
9	3	7	8	2	6	6628.9494	-0.0054
7	3	4	6	2	5	6693.1493	0.0094
10	3	8	9	2	7	6866.5636	0.0159
6	4	3	5	3	2	6907.0498	-0.0014
6	4	2	5	3	3	6926.8673	-0.0010
14	2	13	13	1	12	6956.8621	0.0027
15	2	14	14	1	13	7387.9969	-0.0084
7	4	3	6	3	4	7470.1493	0.0066
8	3	5	7	2	6	7471.3143	-0.0061

Table S32. Measured frequencies and residuals (in MHz) for the rotational transitions of the **181816** isotopologue of the isomer **3w-1** of 1,4-NQ-$(H_2O)_3$.

J'	K'_{-1}	K'_{+1}	J''	K''_{-1}	K''_{+1}	ν_{obs}	ν_{obs}- ν_{calc}
6	1	6	5	0	5	3031.3090	0.0008
7	0	7	6	1	6	3249.3575	0.0096
7	1	7	6	0	6	3425.9496	-0.0013
8	1	8	7	0	7	3836.2145	-0.0073
9	0	9	8	1	8	4199.3033	0.0072
9	1	9	8	0	8	4259.3863	0.0002
7	2	6	6	1	5	4517.0579	0.0041
10	0	10	9	1	9	4658.0340	-0.0032
10	1	10	9	0	9	4691.5806	-0.0010
8	2	7	7	1	6	4811.7553	0.0002
9	2	8	8	1	7	5108.7173	-0.0032
11	0	11	10	1	10	5111.1703	-0.0019
11	1	11	10	0	10	5129.5135	-0.0055
5	3	3	4	2	2	5297.5850	0.0084
10	2	9	9	1	8	5422.7833	-0.0043
5	3	2	4	2	3	5432.9529	-0.0014
12	1	12	11	0	11	5570.8882	-0.0049
6	3	4	5	2	3	5727.9309	-0.0026
4	4	1	3	3	0	5942.2406	-0.0113
13	1	13	12	0	12	6014.2429	-0.0021
6	3	3	5	2	4	6037.8191	-0.0011
7	3	5	6	2	4	6105.2476	-0.0064
12	2	11	11	1	10	6132.7001	0.0027
8	3	6	7	2	5	6429.9624	0.0007
14	0	14	13	1	13	6455.9601	0.0097
14	1	14	13	0	13	6458.7013	-0.0032
5	4	2	4	3	1	6466.6417	-0.0061
5	4	1	4	3	2	6470.8456	-0.0017
13	2	12	12	1	11	6526.6797	0.0033
7	3	4	6	2	5	6702.8415	-0.0047
9	3	7	8	2	6	6708.4586	0.0079
15	0	15	14	1	14	6902.3365	-0.0024
6	4	3	5	3	2	6984.2255	0.0045
6	4	2	5	3	3	7001.0955	0.0068
8	3	5	7	2	6	7452.1513	0.0010
7	4	4	6	3	3	7487.0070	0.0002
7	4	3	6	3	4	7537.4291	0.0064

Table S33. Measured frequencies and residuals (in MHz) for the rotational transitions of the isomer **3w-3** of 1,4-NQ-(H_2O)$_3$.

J'	K'_{-1}	K'_{+1}	J''	K''_{-1}	K''_{+1}	ν_{obs}	ν_{obs}- ν_{calc}
4	1	4	3	0	3	2976.7098	0.0055
5	0	5	4	1	4	3180.5663	-0.0012
5	1	5	4	0	4	3561.2888	0.0036
6	0	6	5	1	5	3890.6438	-0.0065
6	1	6	5	0	5	4148.5430	0.0044
7	1	6	6	2	5	4234.9733	-0.0093
3	3	1	2	2	0	4387.6508	0.0030
3	3	0	2	2	1	4394.1095	-0.0028
5	2	4	4	1	3	4576.0849	-0.0113
7	0	7	6	1	6	4581.1890	0.0000
7	1	7	6	0	6	4745.9877	0.0019
8	1	7	7	2	6	5063.3144	0.0029
4	3	2	3	2	1	5077.1976	-0.0042
4	3	1	3	2	2	5109.8784	-0.0017
6	2	5	5	1	4	5129.6925	0.0024
8	0	8	7	1	7	5255.2206	0.0020
8	1	8	7	0	7	5355.7909	0.0042
7	2	6	6	1	5	5662.1990	0.0040
5	3	2	4	2	3	5842.0596	0.0058
9	1	8	8	2	7	5868.5667	0.0033
9	0	9	8	1	8	5917.1099	-0.0007
9	1	9	8	0	8	5976.3685	0.0034
4	4	1	3	3	0	6006.3735	-0.0035
8	2	7	7	1	6	6182.3328	-0.0024
6	3	4	5	2	3	6377.0818	-0.0040
10	0	10	9	1	9	6570.8450	-0.0019
6	3	3	5	2	4	6602.1520	0.0019
10	1	10	9	0	9	6604.8454	0.0055
9	2	8	8	1	7	6701.3815	-0.0056
5	4	2	4	3	1	6708.8842	-0.0003
7	3	5	6	2	4	6969.5386	-0.0032
11	0	11	10	1	10	7219.4257	-0.0005
11	1	11	10	0	10	7238.5217	-0.0070
11	1	10	10	2	9	7384.1155	-0.0002
6	4	3	5	3	2	7406.6722	0.0057
6	4	2	5	3	3	7418.0624	0.0042
8	3	6	7	2	5	7520.6545	-0.0015
12	0	12	11	1	11	7864.8883	0.0055
12	1	12	11	0	11	7875.4405	-0.0026

Table S34. Measured frequencies and residuals (in MHz) for the rotational transitions of the isomer **3w-4** of 1,4-NQ-$(H_2O)_3$.

J'	K'_{-1}	K'_{+1}	J''	K''_{-1}	K''_{+1}	ν_{obs}	$\nu_{obs}- \nu_{calc}$
6	0	6	5	1	5	2963.8875	0.0004
6	1	6	5	0	5	3118.5651	-0.0010
7	1	6	6	2	5	3413.7516	-0.0047
7	0	7	6	1	6	3469.0837	-0.0014
7	1	7	6	0	6	3552.7411	-0.0011
8	0	8	7	1	7	3958.2919	0.0033
8	1	8	7	0	7	4001.4720	-0.0033
8	1	7	7	2	6	4075.2537	-0.0025
3	3	1	2	2	0	4096.0358	-0.0036
3	3	0	2	2	1	4110.9988	-0.0094
4	2	2	3	1	3	4169.9945	-0.0098
9	0	9	8	1	8	4438.0632	0.0009
7	2	6	6	1	5	4455.8831	-0.0009
9	1	9	8	0	8	4459.6121	-0.0043
9	1	8	8	2	7	4683.8207	0.0047
4	3	1	3	2	2	4706.0773	0.0004
8	2	7	7	1	6	4780.0385	-0.0033
10	0	10	9	1	9	4912.6623	0.0065
5	3	3	4	2	2	5117.8325	-0.0045
9	2	8	8	1	7	5126.5060	-0.0029
10	1	9	9	2	8	5244.6187	0.0187
5	3	2	4	2	3	5341.0676	0.0029
11	0	11	10	1	10	5384.5502	0.0066
11	1	11	10	0	10	5389.5514	0.0028
6	3	4	5	2	3	5542.7969	0.0025
4	4	1	3	3	0	5631.1061	0.0051
4	4	0	3	3	1	5632.4605	0.0009
11	1	10	10	2	9	5769.4574	-0.0006
12	0	12	11	1	11	5855.0743	0.0035
12	1	12	11	0	11	5857.4190	-0.0030
7	3	5	6	2	4	5903.6543	-0.0201
6	3	3	5	2	4	6044.1719	-0.0007
5	4	2	4	3	1	6193.5665	0.0035
5	4	1	4	3	2	6203.1499	-0.0015
8	3	6	7	2	5	6208.7683	-0.0005
13	0	13	12	1	12	6324.9463	0.0120
12	2	11	11	1	10	6349.1163	-0.0054
9	3	7	8	2	6	6472.1886	-0.0080
10	3	8	9	2	7	6712.8075	0.0178
6	4	3	5	3	2	6741.9671	-0.0026
13	1	12	12	2	11	6756.9809	-0.0133
6	4	2	5	3	3	6780.2765	0.0068
14	0	14	13	1	13	6794.4719	-0.0097
13	2	12	12	1	11	6798.0635	-0.0014
5	5	1	4	4	0	7159.7622	0.0117
5	5	0	4	4	1	7159.8546	0.0013

14	1	13	13	2	12	7235.2125	0.0030
7	4	4	6	3	3	7260.5379	-0.0001
7	4	3	6	3	4	7373.4541	-0.0010
6	5	1	5	4	2	7727.6433	0.0074
8	4	5	7	3	4	7728.6189	-0.0114

Table S35. B3LYP-D3BJ/6-311++G(d,p) cartesian coordinates for isomer **1w-1** of 1,4-NQ-H_2O.

Centre number	Atomic Number	Coordinates (Å)		
		X	Y	Z
1	6	-0.332337	-0.648400	0.004673
2	6	1.141596	-0.459365	0.011305
3	6	1.661914	0.926693	0.009859
4	6	0.839500	1.985069	0.006330
5	6	-0.635730	1.847503	0.001766
6	6	-1.191360	0.464157	0.000368
7	6	-0.865606	-1.938611	0.002143
8	6	-2.244159	-2.119960	-0.004207
9	6	-3.097235	-1.014384	-0.008153
10	6	-2.573106	0.274076	-0.005916
11	8	1.908621	-1.416683	0.016445
12	8	-1.352159	2.834667	-0.000627
13	8	4.563647	-0.424484	-0.055373
14	1	-0.186156	-2.781386	0.005045
15	1	-2.656607	-3.121914	-0.006312
16	1	-4.171027	-1.159780	-0.013164
17	1	-3.216083	1.145223	-0.009200
18	1	1.213389	3.002810	0.006168
19	1	2.741815	1.029441	0.014664
20	1	5.309927	-0.960889	0.224715
21	1	3.783006	-1.002175	-0.014485

Table S36. B3LYP-D3BJ/6-311++G(d,p) cartesian coordinates for isomer **2w-1** of 1,4-NQ-$(H_2O)_2$.

Centre number	Atomic Number	Coordinates (Å)		
		X	Y	Z
1	6	1.412180	-1.919451	-0.018292
2	6	0.843768	-0.644145	-0.009460
3	6	1.672501	0.490937	0.004538
4	6	3.058571	0.338651	0.010543
5	6	3.617742	-0.935165	0.002127
6	6	2.795164	-2.063401	-0.012593
7	6	-0.634547	-0.493946	-0.015546
8	6	-1.195537	0.873078	-0.012129
9	6	-0.399578	1.952630	0.003422
10	6	1.078042	1.858465	0.012025
11	8	1.767957	2.864397	0.023977
12	8	-1.368328	-1.479622	-0.021472
13	8	-4.165827	-1.616283	-0.023669
14	8	-4.425110	1.135030	0.040951
15	1	3.677360	1.227087	0.021577
16	1	-0.805441	2.958031	0.010313
17	1	-2.278348	0.968859	-0.019724
18	1	0.756414	-2.780636	-0.030464
19	1	3.234696	-3.053751	-0.019896
20	1	4.695102	-1.051369	0.006643
21	1	-3.189399	-1.639076	-0.038487
22	1	-4.436536	-2.237156	0.658464
23	1	-4.525334	0.162837	0.033794
24	1	-5.087889	1.471083	-0.568329

Table S37. B3LYP-D3BJ/6-311++G(d,p) cartesian coordinates for isomer **2w-2** of 1,4-NQ-$(H_2O)_2$.

Centre number	Atomic Number	Coordinates (Å)		
		X	Y	Z
1	6	2.728523	-0.128776	0.012052
2	6	2.623561	-1.608547	-0.032917
3	6	1.456445	0.642673	0.012145
4	6	1.442090	-2.238024	-0.064790
5	6	0.213496	-0.020716	-0.025874
6	6	0.158753	-1.501507	-0.055453
7	8	3.823372	0.408194	0.046003
8	6	1.495870	2.035740	0.048250
9	6	0.311273	2.768395	0.046384
10	6	-0.919598	2.113190	0.005968
11	6	-0.966149	0.723873	-0.030914
12	8	-0.894376	-2.133310	-0.068584
13	1	2.461129	2.525421	0.077985
14	1	3.565811	-2.144907	-0.036395
15	1	1.360363	-3.318647	-0.095344
16	1	-1.923890	0.229947	-0.065980
17	1	-1.853026	2.662190	0.003961
18	1	0.348895	3.851092	0.075784
19	1	-4.900390	1.280379	-0.700841
20	8	-4.192432	1.125162	-0.069839
21	1	-4.140701	0.156183	0.024088
22	8	-3.669582	-1.637618	0.136441
23	1	-2.723871	-1.852591	0.035901
24	1	-3.995761	-2.206294	0.839576

Table S38. B3LYP-D3BJ/6-311++G(d,p) cartesian coordinates for isomer **3w-1** of 1,4-NQ-$(H_2O)_3$.

Centre number	Atomic Number	Coordinates (Å)		
		X	Y	Z
1	6	-2.236837	-1.786448	0.006987
2	6	-1.383732	-0.681976	-0.033867
3	6	-1.915725	0.618283	0.018988
4	6	-3.295195	0.798494	0.110445
5	6	-4.139932	-0.306479	0.150026
6	6	-3.611493	-1.597885	0.098543
7	6	0.083497	-0.883745	-0.129164
8	6	0.943185	0.310934	-0.163358
9	6	0.442227	1.553497	-0.110947
10	6	-1.013843	1.808410	-0.019827
11	8	-1.449892	2.946536	0.019769
12	8	0.568882	-2.014299	-0.175692
13	8	3.309412	-2.425606	-0.138623
14	8	4.716154	-0.169767	0.543164
15	8	3.521479	2.229713	-0.210685
16	1	-3.683928	1.808288	0.149735
17	1	1.104282	2.411026	-0.135017
18	1	2.010903	0.149802	-0.236880
19	1	-1.805913	-2.778728	-0.033165
20	1	-4.273217	-2.455310	0.130432
21	1	-5.211738	-0.163492	0.221363
22	1	2.333534	-2.342010	-0.165625
23	1	3.553543	-3.025207	-0.848974
24	1	4.999599	-0.306986	1.451390
25	1	4.262012	-0.997019	0.276275
26	1	4.011703	1.430250	0.063888
27	1	4.138038	2.738246	-0.743848

Table S39. B3LYP-D3BJ/6-311++G(d,p) cartesian coordinates for isomer **3w-3** of 1,4-NQ-$(H_2O)_3$.

Centre number	Atomic Number	Coordinates (Å)		
		X	Y	Z
1	6	-1.374494	-1.967002	-0.081133
2	6	-0.857896	-0.697362	-0.339992
3	6	-1.663123	0.437887	-0.155299
4	6	-2.976539	0.291272	0.288298
5	6	-3.486817	-0.977387	0.544205
6	6	-2.686554	-2.106352	0.358227
7	6	0.546502	-0.549708	-0.787970
8	6	1.034971	0.808280	-1.100053
9	6	0.264227	1.889397	-0.936776
10	6	-1.124560	1.798940	-0.429538
11	8	-1.789779	2.807997	-0.264874
12	8	1.282626	-1.525305	-0.927998
13	8	0.943565	0.437093	1.925869
14	8	3.707170	0.464873	1.546046
15	8	3.956454	-0.957474	-0.799469
16	1	-3.578414	1.180322	0.428475
17	1	0.618834	2.890638	-1.153017
18	1	2.056763	0.877573	-1.455571
19	1	-0.734789	-2.828239	-0.225544
20	1	-3.087265	-3.093127	0.558307
21	1	-4.508073	-1.088786	0.889235
22	1	4.533916	-1.717685	-0.913256
23	1	3.040424	-1.279275	-0.944074
24	1	4.213844	1.279649	1.489438
25	1	3.907735	-0.041796	0.730787
26	1	0.764561	-0.059512	2.728936
27	1	1.917866	0.494996	1.869877

Table S40. B3LYP-D3BJ/6-311++G(d,p) cartesian coordinates for isomer **3w-4** of 1,4-NQ-$(H_2O)_3$.

Centre number	Atomic Number	Coordinates (Å)		
		X	Y	Z
1	6	-1.591711	-2.145368	0.052109
2	6	-1.777067	-0.764091	0.027246
3	6	-0.660683	0.090131	-0.082613
4	6	0.621187	-0.457949	-0.170658
5	6	0.800921	-1.838591	-0.147991
6	6	-0.308434	-2.677875	-0.034501
7	6	-3.154800	-0.210262	0.113850
8	6	-3.296250	1.266314	0.072478
9	6	-2.236437	2.077450	-0.031916
10	6	-0.853233	1.558198	-0.107200
11	8	0.076959	2.357749	-0.185200
12	8	-4.143505	-0.918094	0.212846
13	8	2.846356	2.515234	-0.137185
14	8	4.521499	0.471556	0.607375
15	8	3.976578	-2.113567	-0.246365
16	1	1.479293	0.194497	-0.260898
17	1	-2.331083	3.157038	-0.060512
18	1	-4.311149	1.642757	0.133557
19	1	-2.462952	-2.782491	0.138442
20	1	-0.169461	-3.752501	-0.014655
21	1	1.806238	-2.239281	-0.218771
22	1	3.934810	1.204994	0.329698
23	1	4.724973	0.632014	1.532960
24	1	4.683600	-2.363068	-0.847182
25	1	4.200946	-1.211743	0.058395
26	1	1.871661	2.459345	-0.147841
27	1	3.089067	3.167675	-0.799788
